# Glycoprotein Acetyls and Depression: testing for directionality and potential causality using longitudinal data and Mendelian randomization analyses

**DOI:** 10.1016/j.jad.2023.05.033

**Published:** 2023-05-15

**Authors:** Daisy CP Crick, Eleanor Sanderson, Hannah Jones, Neil Goulding, Maria Carolina Borges, Gemma Clayton, Alice R Carter, Sarah L Halligan, Deborah A Lawlor, Golam M Khandaker, Abigail Fraser

**Affiliations:** 1 Population Health Sciences, Bristol Medical School, University of Bristol, Bristol, UK; 2 MRC Integrative Epidemiology Unit at the University of Bristol, Bristol, UK; 3 Department of Psychology, University of Bath, Bath, UK; 4 Department of Psychiatry and Mental Health, University of Cape Town, South Africa; 5 Department of Psychiatry Stellenbosch University, South Africa; 5 NIHR Bristol Biomedical Research Centre, Bristol, UK; 6 Avon and Wiltshire Mental Health Partnership NHS Trust, Bristol, UK

**Keywords:** Glycoprotein Acetyls, Depression, ALSPAC, Mendelian Randomization, Inflammation

## Abstract

**Background:**

Inflammation is associated with depression, but causality remains unclear. We investigated potential causality and direction of effect between inflammation and depression.

**Methods:**

Using data from the ALSPAC birth cohort (n=4021; 42.18% male), we used multivariable regression to investigate bidirectional longitudinal associations of GlycA and depression and depression symptoms, assessed at ages 18y and 24y.

We used two-sample Mendelian randomization (MR) to investigate potential causality and directionality. Genetic variants for GlycA were obtained from UK Biobank (UKB) (N=115,078); for depression from the Psychiatric Genomics Consortium and UKB (N=500,199); and for depressive symptoms (N=161,460) from the Social Science Genetic Association Consortium. In addition to the Inverse Variance Weighted method, we used sensitivity analyses to strengthen causal inference. We conducted multivariable MR adjusting for body mass index (BMI) due to known genetic correlation between inflammation, depression and BMI.

**Results:**

In the cohort analysis, after adjusting for potential confounders we found no evidence of associations between GlycA and depression symptoms score or *vice versa*. We observed an association between GlycA and depression (OR=1·18, 95% CI: 1·03-1·36).

MR suggested no causal effect of GlycA on depression, but there was a causal effect of depression on GlycA (mean difference in GlycA = 0·09; 95% CI: 0·03-0·16), which was maintained in some, but not all, sensitivity analyses.

**Limitations:**

The GWAS sample overlap could incur bias.

**Conclusion:**

We found no consistent evidence for an effect of GlycA on depression. There was evidence that depression increases GlycA in the MR analysis, but this may be confounded/mediated by BMI.

## Introduction

Major Depressive Disorder (MDD) is a prevalent psychiatric disorder and a leading cause of disease burden (Smith, 2014) with a lifetime prevalence of 5-17% (Bains & Abdijadid, 2022). The identification of causal risk factors for MDD could provide novel targets for both prevention and treatment.

An association between inflammation and depression has been identified in cross-sectional and meta-analyses of case/control studies (Howren et al., 2009; Milaneschi et al., 2021b; Osimo et al., 2020). Despite this, reverse causality cannot be ruled out because in both study designs, the exposure and outcome are measured concurrently. Comparatively, prospective cohort studies (which are better able to evaluate temporality/reverse causality) have found evidence of a bi-directional relationship between depression and inflammation (Copeland et al., 2012; Gimeno et al., 2009; Khandaker et al., 2018; Khandaker et al., 2014; Zalli et al., 2016). Although unmeasured confounding may still bias these results, further support for this bidirectional association has come from randomised control trials (RCTs) which are said to be the “gold standard” for epidemiological evidence (Bonaccorso et al., 2001; Capuron et al., 2002; Capuron et al., 2000; Capuron et al., 2001; Köhler et al., 2014; Wittenberg et al., 2020). For example, an RCT found that an interferon-alpha (IFN-α) based treatment for cancer raises serum-based cytokine levels, which in turn predict the development of depressive symptoms (Bonaccorso et al., 2001). However, other factors could drive this association such as the treatment causing lower feelings of wellbeing and hence an increase in symptoms of depression.

Mendelian Randomisation (MR) (Richmond & Davey Smith, 2022; Sanderson et al., 2022) aims to estimate the causal effect of an exposure on an outcome, by utilising the random assortment of genetic variants from parents to offspring (Davey Smith & Ebrahim, 2003). MR has differing and unrelated sources of bias compared to multivariable regression analyses. Integrating results from different methods (triangulation) can enhance causal inference (Lawlor et al., 2017). A description of the assumptions underlying MR is provided in the supplement.

MR studies have begun to investigate the causal effect of inflammation on depressive symptoms (Milaneschi et al., 2021b; Ye et al., 2021). Ye et al. (2021) found that genetically predicted higher CRP concentrations had a protective effect on depression and anxiety. In contrast, genetically predicted higher interleukin-6 (IL-6) levels increased risk of fatigue and sleep problems, but had no effect on overall depression score (Milaneschi et al., 2021b). Other MR studies have reported positive causal effects of soluble IL-6 receptor (sIL-6R) on depression (Kelly et al., 2021) and IL-6 activity increased risk of depressive symptoms (Ye et al., 2021). Despite this, there are a limited number of SNPs associated with circulating levels/concentrations of IL-6 at genome-wide significance that can be used as instrumental variables (IVs; N≤ 2) (Ahluwalia et al., 2021). This makes interpretation of results difficult as the IL-6 IVS sometimes reflect soluble IL-6 receptors (e.g. (Kelly et al., 2021)) rather than circulating IL-6 levels.

Existing research has focused on the IL-6 cytokine and the acute-phase protein CRP (production stimulated by IL-6 levels). Although, archetypal inflammatory markers, both IL-6 and CRP also associate with anti-inflammatory roles within the immune system (Del Giudice et al., 2018). Additionally, both biomarkers show daily fluctuations (Mills et al., 2009; Nilsonne et al., 2016) and respond to short-term stressors (Brydon et al., 2004; Edwards et al., 2006; Gouin et al., 2012; Heesen et al., 2002; Miller et al., 2005; Suarez et al., 2006), suggesting that they are not ideal markers of chronic inflammation (Del Giudice & Gangestad, 2018). Using biomarkers that better index chronic inflammation may help understand the directionality and mechanisms underlying the association between depression and inflammation.

Glycoprotein Acetyls (GlycA) is a nuclear magnetic resonance (NMR) biomarker of inflammation which summarises the signals originating from glycan groups of certain acute-phase glycoproteins. GlycA is elevated in patients with a diverse range of inflammation-linked chronic conditions (Würtz et al., 2012; Würtz et al., 2014) and due to its composite nature (Scott et al., 2015) displays long-term stability (Connelly et al., 2017). Therefore, GlycA provides an overall index of inflammation in the body (Soininen et al., 2015; Würtz et al., 2017) and is likely to be a superior marker of chronic systemic inflammation compared to CRP or IL-6 (Collier et al., 2019; Ritchie et al., 2015).

Here we aim to understand the bidirectional relationship between chronic systemic inflammation measured by GlycA and depression/depressive symptoms. We investigate longitudinal associations using data from a population-based prospective UK birth cohort (Boyd et al., 2013; Fraser et al., 2013; Northstone et al., 2019) and the potential causal effect using bidirectional MR.

## Methods

The Avon Longitudinal Study of Parents and Children (ALSPAC) birth cohort recruited a total of 14,541 pregnant women residing in the former county of Avon; in South-West England (Boyd et al., 2013; Fraser et al., 2013; Northstone et al., 2019). Additional information on ALSPAC is presented in the supplementary file. To be eligible for inclusion in our current study, participants had to have one measure of GlycA and a completed Short Moods and Feelings Questionnaire (SMFQ) at 18y or 24y (N=4021). The participant flowchart is presented in supplementary Figure 1.

We used the SMFQ (Eyre et al., 2021; Thabrew et al., 2018; Thapar & McGuffin, 1998; Turner et al., 2014) administered at mean ages 18y and 24y in ALSPAC as the primary measure. We used the Clinical Interview Schedule-Revised (CIS-R) as a secondary measure (Brugha et al., 1999; Lewis et al., 1992). The CIS-R is a diagnostic tool for depression and can be used to assign International Classification of Diseases-10 (ICD-10) diagnoses of depression (Brugha et al., 1999; Lewis et al., 1992). Here we define the outcome as whether or not an individual was classified as having any form of depression (from mild through to severe).

Plasma GlycA was quantified as part of a high-throughput proton (1H) Nuclear Magnetic Resonance (NMR) metabolomic trait platform at mean ages 18y and 24y (Würtz et al., 2017). Participants fasted overnight (or >6 hours if being seen in the afternoon) before attending the clinic.

Mother’s highest education qualification, participant’s age, smoking status, drinking status, BMI and ethnicity were used as covariates for this analysis. These variables were determined *a priori* based on known or plausible causes of both systemic inflammation and depression.

Further details about all the measures used and sample storage are presented in the supplement.

### Statistical analysis

Multivariable linear regression examined prospective associations between SMFQ scores and GlycA levels and vice versa (exposure at 18y and outcome at 24y). Results were presented sex combined (all p-values for sex interaction ≥ 0·822). To account for multiple testing, we applied a Bonferroni correction (adjusted level of significance=0.013). Information on how the threshold was chosen is presented in the supplement. GlycA levels and SMFQ scores were logged and z-scored to allow comparison between both directions of association.

We used multiple imputation (MI) to impute missing exposure, outcome and confounder data in the eligible sample, creating 25 datasets with 250 iterations. This aimed to balance computational efficiency while ensuring consistency of results (Spratt et al., 2010). The main and auxiliary variables are listed in supplementary table 1. Estimates were combined using Rubin’s rules (Rubin & Schenker, 1991; Spratt et al., 2010; Sterne et al., 2009).

### Univariable bidirectional two-sample Mendelian Randomisation

We used summary-level data from European population GWAS of GlycA (Clayton et al., 2022), MDD (Howard et al., 2019) and depressive symptom (Okbay et al., 2016). The GlycA GWAS was undertaken in UK Biobank data (UKB), the MDD GWAS (referred to as depression onwards) was from the Psychiatric Genomics Consortium (PGC) and the depressive symptoms GWAS was from the Social Science Genetic Association Consortium (SSGAC).

#### Instrument Selection

Genome Wide significant SNPs (P< 5·0 x10-8) were selected from the previously GWAS as instrumental variables for GlycA, depression and depressive symptoms. The datasets were harmonised, aligning the genetic association for exposure and outcome on the effect allele using the effect allele frequency. Palindromic SNPs with allele frequencies of above 0·42 were considered strand ambiguous and removed. SNPs that were unavailable in the outcome datasets were replaced by proxy SNPs at R^2^>0·80. SNPs that had a minor allele frequency of 0·01 or less were excluded.

Following harmonization, there were 50 SNPs in the analysis investigating the effect of GlycA levels on depression (hereafter GlycA-depression), 40 SNPs in the analysis investigating the effect of GlycA levels on depressive symptoms (hereafter GlycA-depressive symptoms) and 47 SNPs in the analysis investigating the effect of depression on GlycA levels (hereafter depression-GlycA). Full details of the instrument selection process are presented in the supplementary materials. The GWAS of depressive symptoms identified only two genome-wide significant SNPs and we therefore did not use depressive symptoms as an exposure. Harmonized genetic association data for selected SNPs are presented in supplementary tables 2-4 respectively.

#### Main analyses

The inverse variance weighted method (IVW method) (Lawlor et al., 2008) was used to estimate effects in the primary analysis and assumes that there is no directional horizontal pleiotropy. MR-Egger, weighted median and weighted mode methods were used as sensitivity analyses. Consistency in results between methods strengthens causal inference because they make different assumptions about pleiotropy (see supplement), whereas divergent results suggest a violation of the third MR assumption (Bowden et al., 2015; Bowden et al., 2016; Hemani et al., 2018). We reported F-statistics as a measure of instrument strength and investigated MR assumptions using: Cochran’s Q, MR-Egger intercept, Radial MR and MR Pleiotropy Residual Sum and Outlier global test (MR-PRESSO). Results are presented post-Steiger filtering. See supplementary table 5 for a description of the MR methods and sensitivity analyses used. A post-hoc estimation of statistical power for each analysis was calculated (Brion et al., 2013) (supplementary table 6). To account for multiple testing, we applied a Bonferroni correction (adjusted level of significance=0.017). This was to account for the three different analyses that were investigated: GlycA-Depression, Depression-GlycA and GlycA-depressive symptoms.

#### Further Analyses

MR-Lap corrects for sample overlap, weak instrument bias and winner’s curse (Mounier & Kutalik, 2021). It was used for the GlycA-depressive symptoms analysis only because both the exposure and outcome must be continuous. A description of MR-Lap is given in the supplement.

As a quality assurance test of the methodology and data sources we replicated our analyses using IL-6 as the biomarker of inflammation. We aimed to replicate previous null findings of the bidirectional relationship between IL-6 and depression (Brunoni et al., 2020; Chu et al., 2021; Gimeno et al., 2009; Milaneschi et al., 2021b; Osimo et al., 2019; Perry et al., 2021). Data sources, SNP selection and method of analysis are described in the supplement.

### Multivariable Mendelian Randomisation

Multivariable Mendelian Randomization (MVMR) is an extension of MR that estimates the direct causal effect of multiple exposures on an outcome (Sanderson et al., 2019) and can be used to account for pleiotropic pathways. Due to the proinflammatory role of adipose tissue (Fantuzzi, 2005) and evidence of its bidirectional association with depression (Milaneschi et al., 2019), we estimated the causal effects of genetically determined depression on inflammation, independently of BMI. A detailed description of MVMR can be found in the supplement. We used the BMI GWAS with the largest number of SNPs available (https://gwas.mrcieu.ac.uk/; data code: ukb-a-248).

#### Instrument selection

We used the same thresholds for genome-wide significance, LD and palindromic SNPs as in the univariable MR. The depression SNPs (n=302 post-pruning) and BMI SNPs (n=316 post-pruning) were combined, forming a final exposure instrument of 618 SNPs for the depression-GlycA MVMR.

Following harmonization with GlycA, 299 SNPs were available for the MVMR analysis. Full details of the SNP selection process are presented in the supplementary material. We only ran an MVMR for depression and BMI as exposures in relation to the outcome GlycA because we found no evidence of an effect of GlycA on depression or depressive symptoms. Due the exposure suffering from weak instruments, we repeated the MVMR using only the genome-wide associated depression SNPs used in our univariable depression-GlycA MR analysis (depression SNPs = 57) (referred to as restricted MVMR onwards; described in the supplement). There were 49 BMI SNPs after pruning and a final exposure instrument of 99 SNPs for the depression-GlycA MVMR. After harmonization with GlycA, there were 47 SNPs available for the MVMR analysis.

#### Software

Information about the software used is presented in the supplement..

## Results

### ALSPAC cohort study

In ALSPAC, observed mean GlycA levels were 1·21 mmol/L (SD: 0·13) at age 18y and 1·22 mmol/L (SD: 0.17) at age 24y. Median SMFQ scores were 5 (Interquartile range: 3-9) at 18y and 5; IQR: 2-10) at 24y. The proportions of individuals with a CIS-R depression diagnosis were 7% and 10% respectively. Unadjusted and adjusted correlation matrices of the depressive symptoms at age 18y and 24y and GlycA levels at 18y and 24y are presented in supplementary figure 2. The distributions of observed and imputed characteristics at ages 18y and 24y are presented in supplementary table 7.

Results from the multivariable regression using the imputed data are presented in table 1. There was no evidence of crude or adjusted associations between depressive scores and GlycA. There was no evidence of any association between depression at 18y and GlycA at 24y. There was a small positive relationship between GlycA levels at 18y and depression at 24y both before and after adjustment, but this did not survive the Bonferroni correction (p<0.013) (table 2). Results from the complete case analysis are presented in supplementary table 8 and were consistent with the main findings.

## Mendelian randomisation analyses

### Instrument validity

SNP-level F statistics were all >10 in univariable analyses, indicating that IVW estimates were not subject to weak instrument bias. The total variance explained (R^2^) across all SNPs in the univariable MR for GlycA-depression was 4·64%, for GlycA-depressive symptoms was 3·84% and for depression-GlycA was 0.36%. All SNPs passed Steiger filtering which means we can assume that the SNPs explain more variation in the exposure than in the outcome for each analysis. Genetic instruments, sample sizes and instrument F-statistics for each GWAS are presented in supplementary table 9.

#### Potential Causal Effect of Genetically Predicted GlycA on Depression and Depressive Symptoms

**Figure 1:**
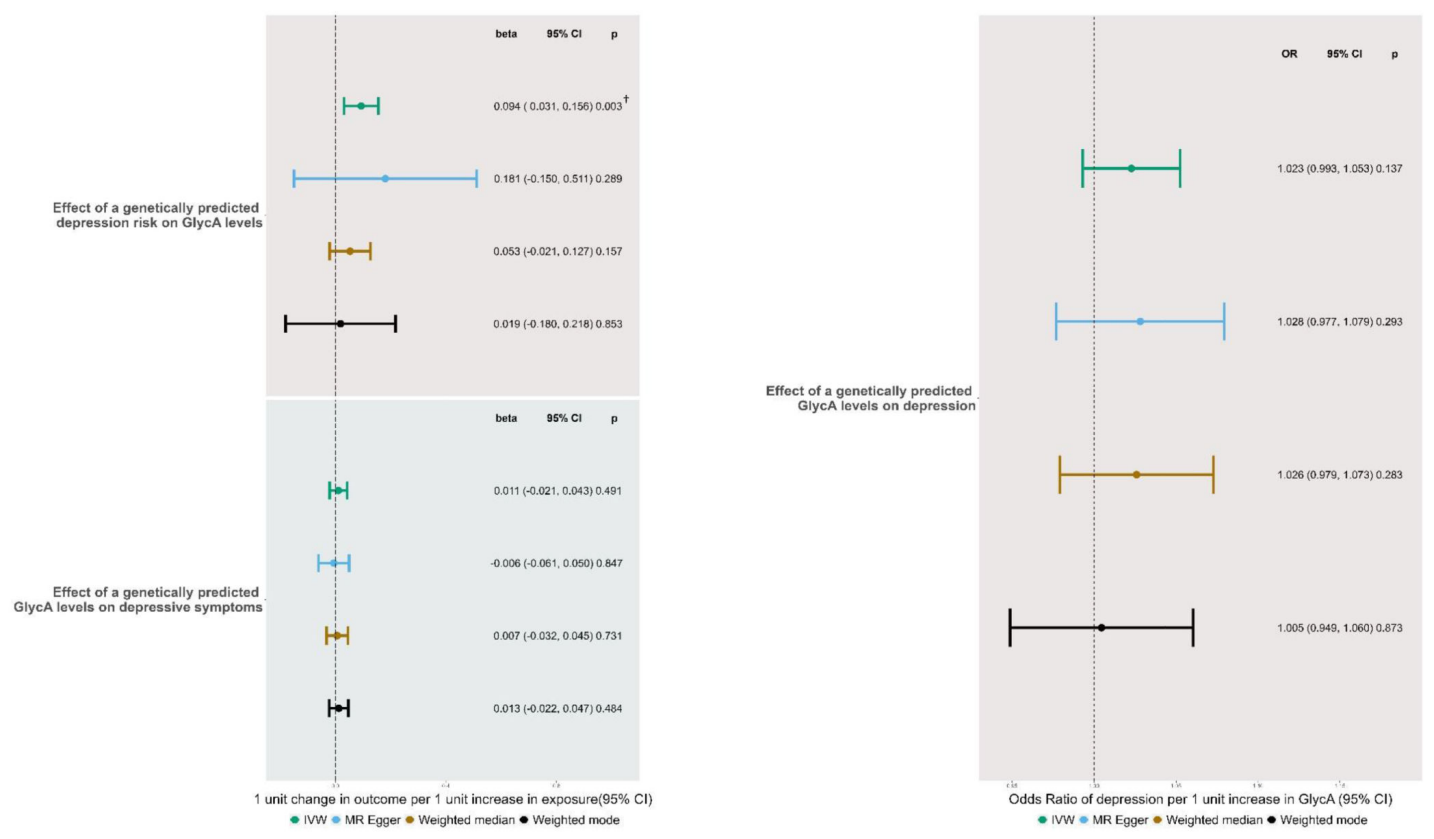
Forest Plot of IVW and sensitivity analyses showing the bidirectional relationship between genetically predicted GlycA on depressive symptoms and MDD. † p<0.017 (Bonferroni correction)

We found no evidence of an effect of genetically predicted GlycA on depression in the MR-IVW analysis (IVW OR = 1·02, 95% CI: 0·99, 1·05). This was consistent across the other MR methods (MR-Egger, weighted median and weighted mode) (figure 1). Neither pleiotropy or heterogeneity were detected and results are presented in the supplement

We found no effect of genetically predicted GlycA on depressive symptoms (IVW mean difference in depressive symptoms per unit change in GlycA =0.01, 95% CI: -0·02, 0·04). This was consistent across the other MR methods (MR-Egger, weighted median and weighted mode) (figure 1). Some pleiotropy was detected and there was evidence of heterogeneity (results presented in the supplement), thus we ran a Radial MR analysis which detects and remove outlier SNPs. Radial MR did not change the results or identify any outliers (results and a further description of radial MR are in the supplement).

#### Potential Causal Effect of Genetically Predicted Depression on GlycA Levels

We found evidence of a positive effect of genetically predicted depression on GlycA levels in the MR-IVW analysis (mean difference in GlycA per unit increase in genetically instrumented depression=0·09, 95% CI=0·03, 0·16) and this withstood the Bonferroni correction for multiple testing (p≤0.017). However, other MR methods yielded estimates consistent with no effect (figure 1). Some pleiotropy was detected and there was evidence of heterogeneity (results presented in the supplement), thus we ran a Radial MR analysis. Radial MR removed 1 SNP but results did not change (see supplement).

### Further Analyses

#### MR-Lap results

There was no effect of GlycA on depressive symptoms (mean difference in depressive symptoms per one-unit increase of GlycA was 0.02, 95% CI: -0.01, 0.04). The non-corrected estimate and the estimate corrected for Winner’s curse, sample overlap and weak instrument bias were similar (mean difference in depressive symptoms per one-unitincrease of GlycA being 0.02; 95% CI: -0.02, 0.05). This suggests that these biases were not affecting our results (p=0.841) (Mounier & Kutalik, 2021).

### IL-6 Analysis

#### Potential Causal Effect of Genetically Predicted IL-6 Levels on Depression

We found no effect of genetically predicted IL-6 on risk of depression (Wald Ratio OR = 1.10, 95% CI: 0.94, 1.28) (supplementary figure 3). The F-statistic suggested that the instrument was weak and Steiger filtering suggested that reverse causation was likely, but due to being the only remaining SNP we did not remove it from the analysis.

#### Potential Causal Effect of Genetically Predicted Depression on IL-6 Levels

We found no effect of genetically predicted depression risk on IL-6 levels in the MR-IVW analysis (mean difference in IL-6 per unit increase in genetically instrumented depression= 0.01, 95% CI = -0.09,0.01). This was consistent across the other MR methods (MR-egger, weighted median and weighted mode) (supplementary figure 3). There was no evidence of pleiotropy or heterogeneity. Further details are provided in the supplement.

Given that these results replicate previous findings in the literature (Brunoni et al., 2020; Chu et al., 2021; Gimeno et al., 2009; Milaneschi et al., 2021b; Osimo et al., 2019; Perry et al., 2021) it provides assurance of the quality in our methodology and data sources.

### MVMR controlling for the effect of BMI

**Figure 2:**
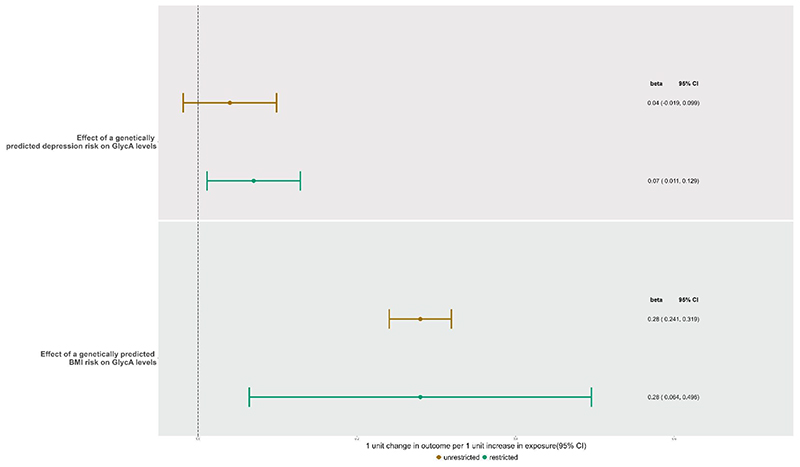
Forest Plot of MVMR-IVW estimates showing the direct effect of genetically predicted GlycA, BMI and MDD on exposures in both the unrestricted and restricted model.

For the independent effect of depression and BMI on GlycA, the conditional F-statistic (Sanderson et al., 2021) suggested the instruments were strong for BMI (F=35·50) but weak for depression (F=4·88). Genetically predicted depression did not have an effect on GlycA (IVW mean difference in GlycA per unit increase in genetically instrumented depression=0·04, 95% CI: -0·03, 0·11) (figure 2). However, there was evidence of heterogeneity across the combined depression and BMI instruments (MVMR Q statistic: p=5·32x10^-19^) suggesting that there could be pleiotropy in the combined set of SNPS. There was a direct effect of BMI on GlycA (IVW mean difference in GlycA per one unit change in BMI=0·29, 95% CI: 0·25, 0·32).

In the restricted MVMR analysis, the conditional F-statistic suggested the instruments were strong for depression (F=28·75) but weaker for BMI (F=8·69). There was evidence of a direct effect of depression on GlycA (IVW = 0·07; 0·01, 0·13) (figure 2). However, again there was evidence of heterogeneity across the set of instruments (MVMR Q statistic: p=0·001). There was a direct effect of BMI on GlycA (IVW= 0·28; 0·06, 0·51).

## Discussion

We found no effect of GlycA levels on depressive symptoms when using multivariable regression or MR. We did find a positive association between GlycA levels and depression when using multivariable regression, however this did not withstand correction for multiple testing (p<0.013) and no effect was found in the MR analysis. Whilst we did find evidence of an effect of genetically predicted depression on GlycA levels using MR which survived Bonferroni correction (p<0.017), this did not persist in all sensitivity analyses and we did not observe an association in multivariable regression. Furthermore, when we controlled for genetically predicted BMI, through the use of MVMR, we found that the effect attenuated to the null. Weak instrument bias may have driven this attenuation and in the restricted MVMR analysis which corrects for weak instruments, a positive effect of depression on resultant GlycA levels persisted.

### Comparison of results to existing literature

Studies examining the links between inflammation and depression including meta-analyses of case/control studies (e.g.(Osimo et al., 2020)), RCTs (e.g.(Hannestad et al., 2011)), longitudinal studies (e.g. (Khandaker et al., 2014) and cross-sectional studies (e.g. (Anderson et al., 2022; Milaneschi et al., 2021a)) have reported associations between inflammatory markers e.g. CRP, IL-6, TNF-alpha and even markers of macrophage activation (Anderson et al., 2022; Postal et al., 2016; Ye et al., 2021; Zalli et al., 2016) and depression. Khandaker *et al*., found that higher levels of IL-6 in childhood were associated with risk of depression and risk of psychotic episodes in later life in a longitudinal study (Khandaker et al., 2014). We may have not replicated this result because inflammation exposure during sensitive periods (e.g., childhood) could play an important role in depression onset (Nagy & Turecki, 2012). As the earliest measure of GlycA we used was in adolescence, we may have missed this effect. The lack of an association may also be because we investigated measures of inflammation and mental health in late adolescence and early adulthood. Aging is associated with increased inflammation (Franceschi & Campisi, 2014) and duration of exposure to inflammation and/or depression. Therefore, we may have missed key associations that appear in later life and this means that we cannot generalise to older age groups. A prospective cohort study by Huckvale et al. (Huckvale et al., 2020) of 3033 individuals with a mean age of 49.79 years found a positive association between GlycA and depression. It is possible that the difference in age range between Huckvale’s study and our study explains the difference in findings. Yet, another prospective cohort study of individuals with a mean age of 51.5 years investigated the association between GlycA and depression incidence, remission and persistence and found that GlycA did not predict onset of depression (Brunoni et al., 2020). Therefore it is possible that the difference between our study and Huckvale’s study arise from reasons other than age, such as ethnic differences; the majority of individuals included in our study were white, whereas the majority of individuals in Huckvale’s study were African American. Additional reasons we did not replicate previous findings in our multivariable regression analyses could include the different inflammatory markers studied or inflammatory marker assessment e.g. NMR signal versus immunoassay.

The majority of MR studies have focused on the effect of genetically predicted inflammatory levels on depression (e.g. (Kelly et al., 2021; Milaneschi et al., 2021b)). Only few have investigated the reverse association; Perry et al., 2021 found no effect of genetically predicted depression on levels of immunological proteins such as IL-6. Here, we show that there may be an effect of genetically predicted depression risk on GlycA levels. Our observed null effect of GlycA on depression could reflect how the GlycA NMR signal is a composite of acute phase proteins (Otvos et al., 2015; Scott et al., 2015). As such, the different proteins may differentially effect depression giving an overall null result (e.g. CRP has been found to have protective effects on depression) (Ye et al., 2021).

### Strengths and Limitations

Here we investigated the understudied bidirectional association between inflammation and depression using a measure of chronic inflammation. We used two different epidemiological methods to investigate the relationship of interest: 1) a multivariable regression longitudinal analysis using a large-population-based cohort and 2) an MR analysis; both of which can reduce bias from reverse causation. We undertook extensive sensitivity analyses and explored pleiotropic paths via BMI in our MR analyses. Future research should explore the depression-GlycA relationship as our finding could have clinical relevance: depression treatments may need to include anti-inflammatory aspects to combat co-morbid inflammation-related health conditions.

However, ALSPAC suffers from attrition which can introduce selection bias and potentially attenuate the results towards the null. We used multiple imputation to minimise any such bias. The post-hoc power calculations suggests that we had limited statistical power to identify the effects of genetically predicted GlycA on depressive symptoms and depression as statistically significant at alpha=0.05. There was sample overlap between the GlycA and depression GWAS, but the bias incurred through sample overlap is less substantial compared to biases produced by weak instruments or winner’s curse (Mounier & Kutalik, 2021). Thus we used the largest depression GWAS to date and aimed to combat the bias using MR-lap, which found no evidence of biased effects.

## Conclusion

In conclusion, we found that GlycA did not appear to be associated with depressive symptoms score in both multivariable regression or MR analyses. We found a potential causal effect of depression on GlycA levels, but this attenuated to the null when accounting for BMI in the MVMR, although the attenuation appeared to be driven by weak instrument bias. In light of inconsistent evidence regarding the potential associations between inflammation and depression, further work is needed to determine if reported associations are causal or not and indeed whether depression increases inflammation, vice versa, both or perhaps neither.

## Supplementary Material

Supplement

## Figures and Tables

**Table 1:** Bidirectional longitudinal associations between continuous SMFQ and GlycA

		Model 1			Model 2*		
	N	Mean difference per SD increase in exposure(SE)	95% CI	*p*	Mean difference per SD increase in exposure (SE)	95% CI	*p*
GlycA at 18y in relation to SMFQ score at 24y	4021	0.03 (0.02)	-0.01, 0.07	0.11	-0.01 (0.02)	-0.05, 0.04	0.80
SMFQ score at 18y in relation to GlycA levels at 24y	4021	0.03 (0.02)	-0.01, 0.07	0.14	0.02 (0.02)	-0.02, 0.06	0.37

* Adjusted for smoking status, drinking status, age in months at baseline, sex, ethnicity, maternal highest education qualification and BMI at 18y.

**Table 2:** Bi-directional longitudinal association between depression and GlycA

		Model 1			Model 2*		
	N	OR (SE)	95% CI	*p*	OR (SE)	95% CI	*p*
GlycA at 18y in relation to depression at 24y	4021	1.32 (0.10)	1.14, 1.53	<0.05^†‡^	1.20 (0.10)	1.01, 1.42	0.04^†^
		**Mean difference per SD increase in exposure(SE)**	**95% CI**	***p***	**Mean difference per SD increase in exposure (SE)**	**95% CI**	***p***
Depression at 18y in relation to GlycA at 24y	4021	0.12 (0.08)	-0.05, 0.28	0.17	0.06 (0.08)	-0.10, 0.23	0.45

* Adjusted for smoking status, drinking status, age in months at baseline, sex, ethnicity, maternal highest education qualification and BMI at 18y.

† p<0.05

‡ *p*< 0.013 (Bonferroni correction)

## References

[R1] Ahluwalia TS, Prins BP, Abdollahi M, Armstrong NJ, Aslibekyan S, Bain L, Jefferis B, Baumert J, Beekman M, Ben-Shlomo Y, Bis JC (2021). Genome-wide association study of circulating interleukin 6 levels identifies novel loci. Hum Mol Genet.

[R2] Anderson AM, Bhondoekhan F, Curanovic D, Connelly MA, Otvos JD, Post WS, Michos ED, Stosor V, Levine A, Seaberg E (2022). Higher Soluble CD163 in Blood Is Associated With Significant Depression Symptoms in Men With HIV. JAIDS Journal of Acquired Immune Deficiency Syndromes.

[R3] Bains N, Abdijadid S (2022). StatPearls.

[R4] Bonaccorso S, Puzella A, Marino V, Pasquini M, Biondi M, Artini M, Almerighi C, Levrero M, Egyed B, Bosmans E, Meltzer HY (2001). Immunotherapy with interferon-alpha in patients affected by chronic hepatitis C induces an intercorrelated stimulation of the cytokine network and an increase in depressive and anxiety symptoms. Psychiatry Res.

[R5] Bowden J, Davey Smith G, Burgess S (2015). Mendelian randomization with invalid instruments: effect estimation and bias detection through Egger regression. International Journal of Epidemiology.

[R6] Bowden J, Davey Smith G, Haycock PC, Burgess S (2016). Consistent Estimation in Mendelian Randomization with Some Invalid Instruments Using a Weighted Median Estimator. Genetic Epidemiology.

[R7] Boyd A, Golding J, Macleod J, Lawlor DA, Fraser A, Henderson J, Molloy L, Ness A, Ring S, Davey Smith G (2013). Cohort Profile: the ‘children of the 90s’--the index offspring of the Avon Longitudinal Study of Parents and Children. Int J Epidemiol.

[R8] Brion MJ, Shakhbazov K, Visscher PM (2013). Calculating statistical power in Mendelian randomization studies. Int J Epidemiol.

[R9] Brugha TS, Bebbington PE, Jenkins R, Meltzer H, Taub NA, Janas M, Vernon J (1999). Cross validation of a general population survey diagnostic interview: a comparison of CIS-R with SCAN ICD-10 diagnostic categories. Psychological Medicine.

[R10] Brunoni AR, Salum GA, Hoffmann MS, Goulart AC, Barreto SM, Canhada S, Carvalho AF, Koyanagi A, Calice-Silva V, Lotufo PA (2020). Prospective associations between hsCRP and GlycA inflammatory biomarkers and depression: The Brazilian longitudinal study of adult health (ELSA-Brasil). Journal of Affective Disorders.

[R11] Brydon L, Edwards S, Mohamed-Ali V, Steptoe A (2004). Socioeconomic status and stress-induced increases in interleukin-6. Brain Behav Immun.

[R12] Capuron L, Gumnick JF, Musselman DL, Lawson DH, Reemsnyder A, Nemeroff CB, Miller AHJN (2002). Neurobehavioral effects of interferon-α cancer patients: phenomenology and paroxetine responsiveness of symptom dimensions.

[R13] Capuron L, Ravaud A, Dantzer RJJoCO (2000). Early depressive symptoms in cancer patients receiving interleukin 2 and/or interferon alfa-2b therapy.

[R14] Capuron L, Ravaud A, Gualde N, Bosmans E, Dantzer R, Maes M, Neveu PJ (2001). Association between immune activation and early depressive symptoms in cancer patients treated with interleukin-2-based therapy. Psychoneuroendocrinology.

[R15] Chu AL, Hickman M, Steel N, Jones PB, Davey Smith G, Khandaker GM (2021). Inflammation and Depression: A Public Health Perspective. Brain, Behavior, and Immunity.

[R16] Clayton GL, Borges MC, Lawlor DA (2022). From menarche to menopause: the impact of reproductive factors on the metabolic profile of over 65,000 women. medRxiv.

[R17] Collier F, Ellul S, Juonala M, Ponsonby A-L, Vuillermin P, Saffery R, Burgner D (2019). Glycoprotein acetyls (GlycA) at 12 months are associated with high-sensitivity C-reactive protein and early life inflammatory immune measures. Pediatric Research.

[R18] Connelly MA, Otvos JD, Shalaurova I, Playford MP, Mehta NN (2017). GlycA, a novel biomarker of systemic inflammation and cardiovascular disease risk. Journal of Translational Medicine.

[R19] Copeland WE, Shanahan L, Worthman C, Angold A, Costello EJ (2012). Cumulative depression episodes predict later C-reactive protein levels: a prospective analysis. Biological psychiatry.

[R20] Davey Smith G, Ebrahim S (2003). ‘Mendelian randomization’: can genetic epidemiology contribute to understanding environmental determinants of disease?. International journal of epidemiology.

[R21] Del Giudice M, Gangestad SW (2018). Rethinking IL-6 and CRP: Why they are more than inflammatory biomarkers, and why it matters. Brain Behav Immun.

[R22] Del Giudice M, Gangestad SWJB, behavior immunity (2018). Rethinking IL-6 and CRP: Why they are more than inflammatory biomarkers, and why it matters. Brain, Behavior, and Immunity.

[R23] Edwards KM, Burns VE, Ring C, Carroll D (2006). Sex differences in the interleukin-6 response to acute psychological stress. Biol Psychol.

[R24] Eyre O, Bevan Jones R, Agha SS, Wootton RE, Thapar AK, Stergiakouli E, Langley K, Collishaw S, Thapar A, Riglin L (2021). Validation of the short Mood and Feelings Questionnaire in young adulthood. Journal of Affective Disorders.

[R25] Fantuzzi G (2005). Adipose tissue, adipokines, and inflammation. Journal of Allergy and clinical immunology.

[R26] Franceschi C, Campisi J (2014). Chronic inflammation (inflammaging) and its potential contribution to age-associated diseases. J Gerontol A Biol Sci Med Sci.

[R27] Fraser A, Macdonald-Wallis C, Tilling K, Boyd A, Golding J, Davey Smith G, Henderson J, Macleod J, Molloy L, Ness A, Ring S (2013). Cohort Profile: the Avon Longitudinal Study of Parents and Children: ALSPAC mothers cohort. Int J Epidemiol.

[R28] Gimeno D, Kivimäki M, Brunner EJ, Elovainio M, De Vogli R, Steptoe A, Kumari M, Lowe GDO, Rumley A, Marmot MG, Ferrie JE (2009). Associations of C-reactive protein and interleukin-6 with cognitive symptoms of depression: 12year follow-up of the Whitehall II study. Psychological Medicine.

[R29] Gouin J-P, Glaser R, Malarkey WB, Beversdorf D, Kiecolt-Glaser JJHP (2012). Chronic stress, daily stressors, and circulating inflammatory markers. Health Psychology.

[R30] Hannestad J, DellaGioia N, Bloch M (2011). The effect of antidepressant medication treatment on serum levels of inflammatory cytokines: a meta-analysis. Neuropsychopharmacology.

[R31] Heesen C, Schulz H, Schmidt M, Gold S, Tessmer W, Schulz KH (2002). Endocrine and cytokine responses to acute psychological stress in multiple sclerosis. Brain Behav Immun.

[R32] Hemani G, Bowden J, Davey Smith G (2018). Evaluating the potential role of pleiotropy in Mendelian randomization studies. Human Molecular Genetics.

[R33] Howard DM, Adams MJ, Clarke TK, Hafferty JD, Gibson J, Shirali M, Coleman JRI, Hagenaars SP, Ward J, Wigmore EM, Alloza C (2019). Genome-wide meta-analysis of depression identifies 102 independent variants and highlights the importance of the prefrontal brain regions. Nat Neurosci.

[R34] Howren MB, Lamkin DM, Suls J (2009). Associations of depression with C-reactive protein, IL-1, and IL-6: a meta-analysis. Psychosomatic Medicine.

[R35] Huckvale S, Reyes S, Kulikova A, Rohatgi A, Riggs KA, Brown ES (2020). An Association Between the Inflammatory Biomarker GlycA and Depressive Symptom Severity. The Journal of Clinical Psychiatry.

[R36] Kelly KM, Smith JA, Mezuk B (2021). Depression and interleukin-6 signaling: A Mendelian Randomization study. Brain Behav Immun.

[R37] Khandaker G, Stochl J, Zammit S, Goodyer I, Lewis G, Jones P (2018). Childhood inflammatory markers and intelligence as predictors of subsequent persistent depressive symptoms: a longitudinal cohort study. Psychological Medicine.

[R38] Khandaker GM, Pearson RM, Zammit S, Lewis G, Jones PB (2014). Association of Serum Interleukin 6 and C-Reactive Protein in Childhood With Depression and Psychosis in Young Adult Life. JAMA Psychiatry.

[R39] Köhler O, Benros ME, Nordentoft M, Farkouh ME, Iyengar RL, Mors O, Krogh J (2014). Effect of Anti-inflammatory Treatment on Depression, Depressive Symptoms, and Adverse Effects. JAMA Psychiatry.

[R40] Lawlor DA, Harbord RM, Sterne JA, Timpson N, Davey Smith G (2008). Mendelian randomization: using genes as instruments for making causal inferences in epidemiology. Statistics in medicine.

[R41] Lawlor DA, Tilling K, Davey Smith G (2017). Triangulation in aetiological epidemiology. International Journal of Epidemiology.

[R42] Lewis G, Pelosi AJ, Araya R, Dunn G (1992). Measuring psychiatric disorder in the community: a standardized assessment for use by lay interviewers. Psychol Med.

[R43] Milaneschi Y, Kappelmann N, Ye Z, Lamers F, Moser S, Jones PB, Burgess S, Penninx BW, Khandaker GMJMP (2021a). Association of inflammation with depression and anxiety: evidence for symptom-specificity and potential causality from UK Biobank and NESDA cohorts.

[R44] Milaneschi Y, Kappelmann N, Ye Z, Lamers F, Moser S, Jones PB, Burgess S, Penninx BWJH, Khandaker GM (2021b). Association of inflammation with depression and anxiety: evidence for symptom-specificity and potential causality from UK Biobank and NESDA cohorts. Molecular Psychiatry.

[R45] Milaneschi Y, Simmons WK, Van Rossum EFC, Penninx BW (2019). Depression and obesity: evidence of shared biological mechanisms. Molecular Psychiatry.

[R46] Miller GE, Rohleder N, Stetler C, Kirschbaum C (2005). Clinical depression and regulation of the inflammatory response during acute stress. Psychosom Med.

[R47] Mills PJ, Natarajan L, Von Känel R, Ancoli-Israel S, Dimsdale JE (2009). Diurnal variability of C-reactive protein in obstructive sleep apnea. Sleep and Breathing.

[R48] Mounier N, Kutalik ZJb (2021). Correction for sample overlap, winner’s curse and weak instrument bias in two-sample Mendelian Randomization.

[R49] Nagy C, Turecki G (2012). Sensitive periods in epigenetics: bringing us closer to complex behavioral phenotypes. Epigenomics.

[R50] Nilsonne G, Lekander M, Åkerstedt T, Axelsson J, Ingre M (2016). Diurnal Variation of Circulating Interleukin-6 in Humans: A Meta-Analysis. PLoS One.

[R51] Northstone K, Lewcock M, Groom A, Boyd A, Macleod J, Timpson N, Wells N (2019). The Avon Longitudinal Study of Parents and Children (ALSPAC): an update on the enrolled sample of index children in 2019. Wellcome Open Res.

[R52] Okbay A, Baselmans BM, De Neve J-E, Turley P, Nivard MG, Fontana MA, Meddens SFW, Linnér RK, Rietveld CA, Derringer JJNg (2016). Genetic variants associated with subjective well-being, depressive symptoms, and neuroticism identified through genome-wide analyses.

[R53] Osimo EF, Baxter LJ, Lewis G, Jones PB, Khandaker GM (2019). Prevalence of low-grade inflammation in depression: a systematic review and meta-analysis of CRP levels. Psychol Med.

[R54] Osimo EF, Pillinger T, Rodriguez IM, Khandaker GM, Pariante CM, Howes OD (2020). Inflammatory markers in depression: A meta-analysis of mean differences and variability in 5,166 patients and 5,083 controls. Brain Behav Immun.

[R55] Otvos JD, Shalaurova I, Wolak-Dinsmore J, Connelly MA, Mackey RH, Stein JH, Tracy RP (2015). GlycA: A Composite Nuclear Magnetic Resonance Biomarker of Systemic Inflammation. Clinical Chemistry.

[R56] Perry BI, Upthegrove R, Kappelmann N, Jones PB, Burgess S, Khandaker GM (2021). Associations of immunological proteins/traits with schizophrenia, major depression and bipolar disorder: A bi-directional two-sample mendelian randomization study. Brain Behav Immun.

[R57] Postal M, Lapa AT, Sinicato NA, de Oliveira Peliçari K, Peres FA, Costallat LTL, Fernandes PT, Marini R, Appenzeller S (2016). Depressive symptoms are associated with tumor necrosis factor alpha in systemic lupus erythematosus. Journal of neuroinflammation.

[R58] Richmond RC, Davey Smith G (2022). Mendelian Randomization: Concepts and Scope. Cold Spring Harbor Perspectives in Medicine.

[R59] Ritchie S, Würtz P, Artika Abraham G, Aki Liam, Sarin A-P, Antti Soininen P, Aalto K, Seppälä I, Raitoharju E, Salmi M, Maksimow M, Männistö S, Kähönen M (2015). The Biomarker GlycA Is Associated with Chronic Inflammation and Predicts Long-Term Risk of Severe Infection. Cell Systems.

[R60] Rubin DB, Schenker N (1991). Multiple imputation in health-care databases: an overview and some applications. Stat Med.

[R61] Sanderson E, Davey Smith G, Windmeijer F, Bowden J (2019). An examination of multivariable Mendelian randomization in the single-sample and two-sample summary data settings. International Journal of Epidemiology.

[R62] Sanderson E, Glymour MM, Holmes MV, Kang H, Morrison J, Munafò MR, Palmer T, Schooling CM, Wallace C, Zhao Q (2022). Mendelian randomization. Nature Reviews Methods Primers.

[R63] Sanderson E, Spiller W, Bowden J (2021). Testing and correcting for weak and pleiotropic instruments in two-sample multivariable Mendelian randomization. Statistics in medicine.

[R64] Scott Würtz P, Artika Abraham G, Aki Liam, Sarin A-P, Antti Soininen P, Aalto K, Seppälä I, Raitoharju E, Salmi M, Maksimow M, Männistö S, Kähönen M, Juonala M, Ripatti S, Lehtimäki T (2015). The Biomarker GlycA Is Associated with Chronic Inflammation and Predicts Long-Term Risk of Severe Infection. Cell Systems.

[R65] Smith K (2014). Mental health: a world of depression. Nature.

[R66] Soininen P, Kangas AJ, Würtz P, Suna T, Ala-Korpela M (2015). Quantitative Serum Nuclear Magnetic Resonance Metabolomics in Cardiovascular Epidemiology and Genetics. Circulation: Cardiovascular Genetics.

[R67] Spratt M, Carpenter J, Sterne JAC, Carlin JB, Heron J, Henderson J, Tilling K (2010). Strategies for Multiple Imputation in Longitudinal Studies. Am J Epidemiol.

[R68] Sterne JA, White IR, Carlin JB, Spratt M, Royston P, Kenward MG, Wood AM, Carpenter JR (2009). Multiple imputation for missing data in epidemiological and clinical research: potential and pitfalls. BMJ.

[R69] Suarez EC, Boyle SH, Lewis JG, Hall RP, Young KH (2006). Increases in stimulated secretion of proinflammatory cytokines by blood monocytes following arousal of negative affect: the role of insulin resistance as moderator. Brain Behav Immun.

[R70] Thabrew H, Stasiak K, Bavin LM, Frampton C, Merry S (2018). Validation of the Mood and Feelings Questionnaire (MFQ) and Short Mood and Feelings Questionnaire (SMFQ) in New Zealand help-seeking adolescents. International Journal of Methods in Psychiatric Research.

[R71] Thapar A, McGuffin P (1998). Validity of the shortened Mood and Feelings Questionnaire in a community sample of children and adolescents: a preliminary research note. Psychiatry Res.

[R72] Turner N, Joinson C, Peters TJ, Wiles N, Lewis G (2014). Validity of the Short Mood and Feelings Questionnaire in late adolescence. Psychol Assess.

[R73] Wittenberg GM, Stylianou A, Zhang Y, Sun Y, Gupta A, Jagannatha PS, Wang D, Hsu B, Curran ME, Khan S, Chen G (2020). Effects of immunomodulatory drugs on depressive symptoms: A mega-analysis of randomized, placebo-controlled clinical trials in inflammatory disorders. Molecular Psychiatry.

[R74] Würtz P, Kangas AJ, Soininen P, Lawlor DA, Davey Smith G, Ala-Korpela M (2017). Quantitative Serum Nuclear Magnetic Resonance Metabolomics in Large-Scale Epidemiology: A Primer on-Omic Technologies. Am J Epidemiol.

[R75] Würtz P, Mäkinen V-P, Soininen P, Kangas AJ, Tukiainen T, Kettunen J, Savolainen MJ, Tammelin T, Viikari JS, Rönnemaa T, Kähönen M, Lehtimäki T, Ripatti S (2012). Metabolic Signatures of Insulin Resistance in 7,098 Young Adults. Diabetes.

[R76] Würtz P, Wang Q, Kangas AJ, Richmond RC, Skarp J, Tiainen M, Tynkkynen T, Soininen P, Havulinna AS, Kaakinen M (2014). Metabolic signatures of adiposity in young adults: Mendelian randomization analysis and effects of weight change. PLoS Medicine.

[R77] Ye Z, Kappelmann N, Moser S, Davey Smith G, Burgess S, Jones PB, Khandaker GM (2021). Role of inflammation in depression and anxiety: Tests for disorder specificity, linearity and potential causality of association in the UK Biobank. EClinicalMedicine.

[R78] Zalli A, Jovanova O, Hoogendijk WJG, Tiemeier H, Carvalho LA (2016). Low-grade inflammation predicts persistence of depressive symptoms. Psychopharmacology.

