# Supplementary material for: Glycoprotein Acetyls and Depression: testing for directionality and potential causality using longitudinal data and Mendelian randomization analyses
Source: J Affect Disord. Author manuscript; Available in PMC 2024 Jan 5. (PMC7615476; doi:10.1016/j.jad.2023.05.033)
Supplement: Supplement [file EMS192904-supplement-Supplement.docx]

***Contents page***

| ***Alspac cohort recruitment*** | ***2*** |
| --- | --- |
| **Depressive symptoms and depression variables** | ***3*** |
| **Sample storage** | ***4*** |
| **Choice of primary measure** | ***5*** |
| **Covariate variables** | ***6*** |
| ***Bonferroni Threshold*** | ***7*** |
| ***Mendelian Randomization*** | ***8*** |
| ***Instrument selection and Data Sources*** | ***9*** |
| ***Assumptions of pleiotropy*** | ***12*** |
| ***Software*** | ***13*** |
| ***Heterogeneity and Pleiotropy*** | ***14*** |
| ***MR-Lap*** | ***15*** |
| ***Multivariable Mendelian randomization*** | ***16*** |
| ***Radial MR*** | ***19*** |
| ***IL-6 Analysis*** | ***20*** |
| ***References*** | ***22*** |
| ***Supplementary tables*** | ***25*** |
| ***Supplementary figures*** | ***42*** |

**Alspac Cohort Recruitment**

Women were recruited if they had an expected delivery date between 1st April 1991 and 31st December 1992. When the oldest children were approximately 7 years of age, an attempt was made to bolster the initial sample with eligible cases who did not originally join the study. The total sample size for analyses using any data collected after the age of seven is therefore 15,454 pregnancies, resulting in 15,589 foetuses. Of these, 14,901 were alive at 1 year of age. The offspring, their mothers and the mother’s partners are regularly followed up.

Please note that the ALSPAC website contains details of all the data that is available through a fully searchable data dictionary and variable search tool http://www.bristol.ac.uk/alspac/researchers/our-data/. The research was conducted under the principles of the Declaration of Helsinki. Ethical approval for the study was obtained from the ALSPAC Ethics and Law Committee and the Local Research Ethics Committee and can be found here: <http://www.bristol.ac.uk/alspac/researchers/research-ethics/>. Informed consent for the use of data collected via questionnaires and clinics was obtained from participants following the recommendations of the ALSPAC Ethics and Law Committee at the time. Consent for biological samples has been collected in accordance with the Human Tissue Act (2004).

Study data were collected and managed using REDCap electronic data capture tools hosted at the University of Bristol. REDCap (Research Electronic Data Capture) is a secure, web-based software platform designed to support data capture for research studies (Harris et al., 2009).

**Depressive symptoms and depression variables**

The SMFQ comprises 13 items relating to depressive symptoms experienced in the last two weeks. Each item is rated on a 3-point scale (0=not true, 1=sometimes true, 2=true), giving a total score of 0 to 26. Higher scores are suggestive of a greater burden of depressive symptoms.

The CIS-R yields a depression diagnosis according to ICD-10 criteria, including levels of mild depression, moderate depression and severe depression. We created a binary variable of 0 (no depression) and 1 (depression present at any level of severity). We used this second measure of depression because although the SMFQ provides information about depressive symptoms, we are also interested in the relationship between inflammation and depression which the CIS-R, a diagnostic tool can provide. Given that the SMFQ demonstrates good discriminatory abilities for identification of depression as measured by the CIS-R (Costantini et al., 2021) and the limited statistical power of the CIS-R binary measure, the SMFQ score was used as a primary outcome.

**Sample Storage**

Plasma samples were stored at -80 °C and slowly thawed in a refrigerator (+4^0^) the night before processing. The samples were mixed and spun in a centrifuge at 3400 x *g* to remove precipitation. There was no other freeze-thaw cycles and samples were analysed within 3–9 months of collection. Further details regarding sample/data processing and NMR analysis have been provided elsewhere (Inouye et al., 2010; Soininen et al., 2009).

**Choice of primary measures**

We chose to use the SMFQ as the primary measure of depressive symptoms because as stated in the article it is a widely validated tool to measure depressive symptoms in clinical and non-clinical participants (Eyre et al., 2021; Thabrew et al., 2018; Thapar & McGuffin, 1998; Turner et al., 2014). Additionally, the SMFQ has performed well in cases and non-cases of MDD at age 25y, supporting its use in our dataset (Eyre et al., 2021). The English version has been translated into Arabic, Spanish and Norwegian and given it is a continuous measure it has greater power than the CIS-R diagnostic tool. Additionally, the SMFQ is freely available and takes approximately three-five minutes to complete (Costello & Angold, 1988). The SMFQ is based on a scoring system where increase in score suggests higher depressive symptoms. There is a recognised clinical cut-off of >12 which equates to depression (Jarbin et al., 2020).

GlycA was chosen as the primary measure of inflammation because we are interested in the association between chronic, systemic inflammation and depression. Given the high rates of stability seen in GlycA (Connelly et al., 2017) we opted for this biomarker over other previously used inflammatory biomarkers such as C-reactive Protein or Interleukin-6. GlycA is one of the 200 metabolic measures that is quantified by NMR spectroscopy. This quantitative metabolomics platform has widespread use (Würtz et al., 2017) and the all-in-one approach means that it is relatively cost-effective. Further, datasets with metabolomic data are likely to have measures of GlycA. Given the novelty of GlycA the clinical meaning of a change in GlycA score is uncertain, however higher levels of GlycA indicate higher levels of systemic chronic inflammation.

**Covariate Variables**

Mother’s highest education qualification self-reported at 8-42 weeks gestation was reported and categorised as: below Ordinary-level (exams taken at age 15–16 years); Vocational or O-level, Advanced-level (exams taken at age 18 years); and university degree or above). Participants’ age (reported in months), smoking status and drinking status (non-smoker, infrequent smoker and frequent smoker) and BMI were reported at ages 18y and 24y. Participants’ ethnicity was recorded and coded as white or non-white. To be classified as an infrequent smoker/drinker, participants had to report smoking/drinking less than once a week. To be classified as a frequent smoker/drinker, participants has to report smoking/drinking more than once a week. BMI was calculated as weight in kilograms divided by height in meters squared, where weight was measured with the use of Tanita scales to the nearest 0.1 kg and height was measured using a Harpenden standiometer to the nearest 0.1 cm.

**Bonferroni Threshold**

A Bonferroni threshold of p<0.013 was chosen given that there were four tests: the primary analysis investigating the bidirectional association between SMFQ and GlycA and the secondary analysis investigating the bidirectional association between the CIS-R and GlycA. This is a conservative threshold given that not all tests are strictly independent

**Mendelian Randomization**

Mendelian Randomization is bound by three assumptions: (i) the genetic variants are statistically strongly associated with the exposure of interest and relevant to the population to which inference is being made (the relevance assumption); (ii) there is no confounding of the SNP-outcome association (the independence assumption); and (iii) any effect of the genetic instrument on the outcome is only via the exposure (the exclusion restriction assumption) (Sanderson et al., 2022) (supplementary figure 4).

**Instrument Selection and data sources**

The differences in number of SNPs available for each analysis is because not all SNPs were available in each GWAS.

***Data sources and SNP Selection for GlycA***

We obtained GlycA summary genetic estimates from a GWAS using UKBB. This was made up of 115,078 males and females from European descent. A total of 61 independent Single Nucleotide Polymorphisms (SNPs) reached genome-wide significance (p<5x10^-8^) and were selected as instrumental variables (IVs). Among the 61 GlycA-associated SNPs, 4 were removed due to linkage disequilibrium (R^2^=0.01) leaving 57 SNPs available for harmonisation.

Among the 57 GlycA-associated SNPS, 46 were available in the depression GWAS and 5 of the missing SNPs were replaced by suitable proxies. Of these remaining SNPs, 1 was excluded for being palindromic with intermediate allele frequencies. This gave 50 SNPs as instrumental variables in the analysis of the effect of genetically predicted GlycA on the occurrence of depression.

When harmonizing the 57 GlycA-associated SNPs with depressive symptoms, 40 SNPs were available in the dataset of depressive symptoms and 2 of the missing SNPs were replaced by suitable proxies. Of these remaining SNPs, 2 were excluded for being palindromic with intermediate allele frequencies. This resulted in 40 SNPs as instrumental variables in the analysis of genetically predicted GlycA on the occurrence of depressive symptoms.

***Data sources and SNP Selection for depression***

The depression GWAS included meta-analysed data from three of the largest GWAS’ of depression: an MDD GWAS using 23andme data (Hyde et al., 2016), an MDD GWAS using UKBB data (Howard et al., 2018) and an MDD GWAS using PGC data (Wray et al., 2018). The meta-analysed data was made up of 807,553 individuals of European-decent (David M Howard et al., 2019). For our analysis, the GWAS from 23andme was removed due to poor MDD outcome definition (23andme use a self-reported MDD measure, compared to PGC and UKBB where MDD is based on the DSM (Diagnostic and Statistical Manual of Mental Disorders) case definition and/or validated clinical questionnaires). Therefore the final sample was made up of 500,199 males and females of European-decent. A total of 50 independent SNPs reached genome-wide significance (p <5 x10^-8^) and of the 50 depression-associated SNPs, 1 was removed due to linkage disequilibrium (R^2^=0.01) leaving 49 SNPs available for harmonisation.

Among the 49 depression-associated SNPs, 2 SNPs were excluded for being palindromic with intermediate allele frequencies, resulting in 47 SNPs as IVs in the analysis of a genetic predisposition to depression on GlycA levels.

***Data sources and SNP Selection for depressive symptoms***

We obtained genetic summary estimates for individuals with depressive symptoms from a GWAS using SSGAC. This was made up of 161,460 individuals of European-decent (Okbay et al., 2016). Only 2 SNPs (p <5 x10^-8^; Linkage disequilibrium r^2^=0.01) were selected as instrumental variables for depressive symptoms. In the MR investigating a genetic liability of depressive symptoms on GlycA no proxy SNPs were required. However, 1 SNP was removed due to being palindromic with intermediate allele frequencies leaving only 1 SNP available for the analysis between depressive symptoms and GlycA. An MR was therefore not run on this association.

**Assumptions of pleiotropy**

The IVW assumes no unbalanced horizontal pleiotropy and forces the intercept to go through zero. In contrast, MR-Egger does not force the line through zero and as a result the intercept gives an indication of the presence of pleiotropy (Bowden et al., 2015)[45]. The weighted median and weighted mode assume that the pleiotropic effect of certain SNPs on the outcome are less likely to converge on a common median or modal estimate. However, the valid SNPs that display no pleiotropic effects will show more uniform and homogenous effects on the exposure and outcome. This makes them more likely to cluster toward the median/modal point estimate (Bowden et al., 2016; Hartwig et al., 2017).

**Software**

Multivariable linear regression analyses were performed using STATA version 17.0. All other analyses were performed in R Software version 4.1.1. Code for data management and statistical analysis has been made available in: [daisycrick/MR_Inflammation_Depression: Investigating the bidirectional association between GlycA and MDD (github.com)](https://github.com/daisycrick/MR_Inflammation_Depression)

**Heterogeneity and Pleiotropy**

***Potential Causal Effect of Genetically Predicted GlycA Levels on Depression***

There was no evidence of directional pleiotropic effects (MR-Egger-intercept -0.0004, p=0∙805), however MR-PRESSO suggested that horizontal pleiotropy was present (p= 0∙232) but identified no outlier SNPs. There was also no heterogeneity detected in either the IVW and MR-Egger analysis (Q=54∙41, p=0∙276 and Q=54∙34, p=0∙246 respectively).

***Potential Causal Effect of Genetically Predicted GlycA Levels on Depressive Symptoms***

There was little evidence of pleiotropic effects suggested by the MR-Egger-intercept (0∙001, p=0∙477). MR-PRESSO found evidence for horizontal pleiotropy (p= 0∙02) but identified no outlier SNPs. Heterogeneity was detected in both the IVW and MR-Egger analysis (Q=60∙11, p=0∙017 and Q=59∙30, p=0∙015 respectively). Radial MR did not change the results or identify any outliers (see Radial MR section).

***Potential Causal Effect of Genetically Predicted Depression on GlycA Levels***

There was little evidence of pleiotropic effects (MR-Egger-intercept -0∙003, p =0∙601) although the MR-Egger regression is imprecisely estimated. MR PRESSO suggested the presence of horizontal pleiotropy (p<0∙001) and detected 1 outlier, but its removal did not alter results (comparison between original and outlier corrected results p=0∙721). There was also evidence of heterogeneity in the IVW and MR-Egger analysis (Q=46∙0 and 45∙0 respectively, both p<0∙001). Radial MR removed 1 SNP but results did not change (see Radial MR section).

**MR-Lap**

MR-Lap is a novel method corrects for sample overlap, weak instrument bias and winner’s curse (Mounier & Kutalik, 2021). If the MR-LAP corrected effect does not significantly differ from the observed effect, then the IVW-MR estimate can be used. If there is a difference between the two estimates, it suggests that the biases are having an effect on the effect estimate and therefore, the corrected effect would be preferred (Mounier & Kutalik, 2021). For this analysis, we used the same GlycA and depressive symptoms GWAS as used in the main analysis. Instrumental variables (IVs) were pruned to the distance threshold of 10,000 Kb with an LD threshold of 0.001, giving 49 IVs as part of the analysis.

**Multivariable Mendelian Randomization**

Multivariable Mendelian Randomization (MVMR) estimates the direct effect of each exposure on the outcome and can be used to account for pleiotropic pathways. In this way the univariable MR can be thought of as the *total* *effect* in an epidemiological mediation model, whereas the MVMR is analogous to the *direct* *effects* of each exposure on the outcome (supplementary figure 5). This analysis adjusts for pleiotropic effects of the exposure SNPs which act via BMI.

Genetic correlations of GlycA and BMI, and depression and BMI (r=0.334 and r=0.086 respectively) were estimated through linkage disequilibrium score regression. This was to check that the variables were genetically correlated and therefore an MVMR was applicable. As described in the main manuscript, we repeated the MVMR using only the depression SNPs identified in the univariable MR (known as the restricted MVMR). This was because in the initial MVMR analysis, after the depression and BMI SNPs were combined, there was a limited number of remaining depression SNPs. This means that many of the depression SNPs were in LD with BMI, suggesting that they may be weak instruments or that many of the depression SNPs were acting through BMI. We did this by merging the 57 depression SNPs with the IVs for BMI, LD clumped the combined data and then harmonised with the GlycA data.

In the MVMR analysis investigating the effect of genetically predicted depression on GlycA levels, while controlling for BMI, 49 of 365 SNPs were removed due to LD or absence from LD reference panel and 32 of the missing SNPs were replaced by suitable proxies. No proxies were needed for the outcome data. During harmonization, 10 SNPs were dropped for being palindromic with intermediate allele frequencies. In the MVMR analysis investigating the effect of genetically predicted GlycA on depression, while controlling for BMI, 69 of the 376 SNPs were removed due to LD with other SNPS or absence from LD reference panel and 2 of the missing BMI SNPs were replaced by suitable proxies. In the GlycA data, 33 of the missing SNPs were replaced by suitable proxies. During harmonization, 10 SNPs were dropped for being palindromic with intermediate allele frequencies.

Heterogeneity of instrument effects was evaluated using a modified form of Cochran’s Q statistic. This assesses horizontal pleiotropy with respect to differences in MVMR estimates across the set of instruments. Any observed heterogeneity is indicative of a violation of the exclusion restriction assumption. In the MVMR there was evidence of heterogeneity across the combined depression and BMI instruments (MVMR Q statistic: p=5∙32x10^-19^) suggesting that there could be pleiotropy in the combined set of SNPS. there was a direct effect of BMI on GlycA (IVW mean difference in GlycA per one unit change in BMI=0∙29, 95% CI: 0∙25, 0∙32). In the restricted MVMR there was evidence of heterogeneity across the set of instruments (MVMR Q statistic: p=0∙001). There was a direct effect of BMI on GlycA (IVW= 0∙28; 0∙06, 0∙51).

Instrument strength was evaluated through a conditional F-statistic (the same conventional instrument strength threshold of 10 can be used) (Sanderson et al., 2021). The F-statistic assessing instrument strength, whether the genetic variants used as instruments are required to strongly associate with their exposures, conditioning on the remaining included exposures. We required the variance-covariance for the effects of the genetic variants on each exposure to test for weak instrument and heterogeneity and therefore assessed the phenotypic co

for the effects of the genetic variants on each exposure to test for weak instrument and heterogeneity and therefore assessed the phenotypic co

**Radial MR**

Radial MR is similar to IVW, but detects and removes outlying variants via a simulation-based approach to then re-estimate the original exposure-outcome relationship. This can reduce bias in MR estimates, but can sometimes lead to over-fitting because the standard error is reduced. Radial plots can help to interpret the validity of the IVW and MR-Egger regression and improve the detection of outliers and data points that are influential in the IVW or MR-Egger analysis.

***Effect of Genetically Predicted GlycA levels on Depressive Symptoms***

No significant outliers were detected and we observed a consistent effect direction as the original MR analysis for Radial IVW (*beta=0.01*; 95% CI -0.02, 0.04; *P* value = 0.491) and Radial MR-Egger (*beta=* -0.002; 95% CI -0.06, 0.06); *P* value =  0.959). Cochran’s Q statistic and the Rucker’s Q statistic indicated heterogeneity (p = 0.017 and p = 0.018 respectively). The radial IVW plot is presented in supplementary figure 6.

***Effect of Genetically Predicted Depression on GlycA levels***

Using Radial-IVW, one outlier was detected and removed (rs7725715, p = 0.001). The corrected effect was consistent with the main analysis (*beta=0.09*; 95% CI 0.03, 0.15; *P* value = 0.002). The same outlier was detected (p=0.001) in the Radial MR-Egger n (*beta=* 0.23; 95% CI -0.25, 0.71]; *P* value =  0.346). It is worth noting that the Radial MR-Egger approach often has wider confidence intervals due to it being statistically relatively inefficient. Cochran’s Q statistic and the Rucker’s Q statistic indicated heterogeneity (p <0.001 for both). The radial IVW plot is presented in supplementary figure 7.

**IL-6 Analysis**

***Data sources and SNP Selection for IL-6***

We obtained IL-6 summary genetic estimates from a 26 cohort GWAS meta-analysis (Ahluwalia et al., 2021). This was the most recent and one of the largest IL-6 GWAS’ to date and made up of 52,654 males and females from European descent. They identified 94 variants that were genome-wide significantly associated with IL-6 levels. However, only 2 SNPs (p <5 x10^-8^; Linkage disequilibrium r^2^=0.01) were selected as instrumental variables due to high levels of LD. In the MR analysis investigating the effect of genetically predicted IL-6 levels on depression one proxy SNP were not available leaving only 1 SNP available for the analysis. Previous analyses using IL-6 exposure IVs has reported a similar issue and we therefore opted to use a Wald ratio for this analysis which will complement previous work (Perry et al., 2021).

We used the same depression GWAS as in the main MR analysis with GlycA and therefore had 49 depression-associated SNPs. Among the 49 depression-associated SNPs, 1 SNP was excluded for being palindromic with intermediate allele frequencies. No proxies were available, resulting in 22 SNPs as IVs in the analysis investigating the effect of genetically predicted depression on IL-6 levels. Harmonized SNPs are presented in Supplementary tables 10-11.

***Heterogeneity and Pleiotropy***

We were only able to investigate the pleiotropic effects and heterogeneity effects in the analysis evaluating the potential causal effect of genetically predicted depression on IL-6 levels. There was no evidence of pleiotropic effects suggested by the MR-Egger-intercept (-0.0001, p=0.988). MR-PRESSO did not detect any outliers and the MR PRESSO Global test found no suggestion of horizontal pleiotropy for the effect of IL-6 on depression (p=0.677). Heterogeneity was not detected in either the IVW and MR-Egger analysis (Q=10, p=1.00 and Q=9, p=1.00 respectively). Of the 22 SNPs, 11 did not pass Steiger filtering. However, the results did not change and we present results with the SNPs removed.

Ahluwalia, T. S., Prins, B. P., Abdollahi, M., Armstrong, N. J., Aslibekyan, S., Bain, L., Jefferis, B., Baumert, J., Beekman, M., Ben-Shlomo, Y., Bis, J. C., Mitchell, B. D., de Geus, E., Delgado, G. E., Marek, D., Eriksson, J., Kajantie, E., Kanoni, S., Kemp, J. P., Lu, C., Marioni, R. E., McLachlan, S., Milaneschi, Y., Nolte, I. M., Petrelis, A. M., Porcu, E., Sabater-Lleal, M., Naderi, E., Seppälä, I., Shah, T., Singhal, G., Standl, M., Teumer, A., Thalamuthu, A., Thiering, E., Trompet, S., Ballantyne, C. M., Benjamin, E. J., Casas, J. P., Toben, C., Dedoussis, G., Deelen, J., Durda, P., Engmann, J., Feitosa, M. F., Grallert, H., Hammarstedt, A., Harris, S. E., Homuth, G., Hottenga, J. J., Jalkanen, S., Jamshidi, Y., Jawahar, M. C., Jess, T., Kivimaki, M., Kleber, M. E., Lahti, J., Liu, Y., Marques-Vidal, P., Mellström, D., Mooijaart, S. P., Müller-Nurasyid, M., Penninx, B., Revez, J. A., Rossing, P., Räikkönen, K., Sattar, N., Scharnagl, H., Sennblad, B., Silveira, A., Pourcain, B. S., Timpson, N. J., Trollor, J., van Dongen, J., Van Heemst, D., Visvikis-Siest, S., Vollenweider, P., Völker, U., Waldenberger, M., Willemsen, G., Zabaneh, D., Morris, R. W., Arnett, D. K., Baune, B. T., Boomsma, D. I., Chang, Y. C., Deary, I. J., Deloukas, P., Eriksson, J. G., Evans, D. M., Ferreira, M. A., Gaunt, T., Gudnason, V., Hamsten, A., Heinrich, J., Hingorani, A., Humphries, S. E., Jukema, J. W., Koenig, W., Kumari, M., Kutalik, Z., Lawlor, D. A., Lehtimäki, T., März, W., Mather, K. A., Naitza, S., Nauck, M., Ohlsson, C., Price, J. F., Raitakari, O., Rice, K., Sachdev, P. S., Slagboom, E., Sørensen, T. I. A., Spector, T., Stacey, D., Stathopoulou, M. G., Tanaka, T., Wannamethee, S. G., Whincup, P., Rotter, J. I., Dehghan, A., Boerwinkle, E., Psaty, B. M., Snieder, H., & Alizadeh, B. Z. (2021). Genome-wide association study of circulating interleukin 6 levels identifies novel loci. *Hum Mol Genet*, *30*(5), 393-409. <https://doi.org/10.1093/hmg/ddab023>

Bowden, J., Davey Smith, G., & Burgess, S. J. I. j. o. e. (2015). Mendelian randomization with invalid instruments: effect estimation and bias detection through Egger regression. *44*(2), 512-525.

Bowden, J., Davey Smith, G., Haycock, P. C., & Burgess, S. J. G. e. (2016). Consistent estimation in Mendelian randomization with some invalid instruments using a weighted median estimator. *40*(4), 304-314.

Connelly, M. A., Otvos, J. D., Shalaurova, I., Playford, M. P., & Mehta, N. N. (2017). GlycA, a novel biomarker of systemic inflammation and cardiovascular disease risk. *J Transl Med*, *15*(1), 219. <https://doi.org/10.1186/s12967-017-1321-6>

Costantini, I., Kwong, A. S., Smith, D., Lewcock, M., Lawlor, D. A., Moran, P., Tilling, K., Golding, J., & Pearson, R. M. (2021). Locus of control and negative cognitive styles in adolescence as risk factors for depression onset in young adulthood: Findings from a prospective birth cohort study. *Frontiers in psychology*, *12*, 599240.

Costello, E. J., & Angold, A. (1988). Scales to assess child and adolescent depression: checklists, screens, and nets. *Journal of the American Academy of Child & Adolescent Psychiatry*, *27*(6), 726-737.

Davies, N. M., Holmes, M. V., & Davey Smith, G. (2018). Reading Mendelian randomisation studies: a guide, glossary, and checklist for clinicians. *BMJ*, *362*, k601. <https://doi.org/10.1136/bmj.k601>

Eyre, O., Bevan Jones, R., Agha, S. S., Wootton, R. E., Thapar, A. K., Stergiakouli, E., Langley, K., Collishaw, S., Thapar, A., & Riglin, L. (2021). Validation of the short Mood and Feelings Questionnaire in young adulthood. *Journal of Affective Disorders*, *294*, 883-888. <https://doi.org/10.1016/j.jad.2021.07.090>

Harris, P. A., Taylor, R., Thielke, R., Payne, J., Gonzalez, N., & Conde, J. G. (2009). Research electronic data capture (REDCap)--a metadata-driven methodology and workflow process for providing translational research informatics support. *J Biomed Inform*, *42*(2), 377-381. <https://doi.org/10.1016/j.jbi.2008.08.010>

Hartwig, F., Davey Smith, G., & Bowden, J. J. I. J. o. E. (2017). summary data Mendelian randomisation via the zero modal pleiotropy assumption. International Journal of Epidemiology, 46 (6), 1985-1998.[dyx102]. <https://doi>. org/10.1093/ije/dyx102. *1*, 14.

Hemani, G., Tilling, K., & Davey Smith, G. (2017). Orienting the causal relationship between imprecisely measured traits using GWAS summary data. *PLOS Genetics*, *13*(11), e1007081. <https://doi.org/10.1371/journal.pgen.1007081>

Howard, D. M., Adams, M. J., Clarke, T.-K., Hafferty, J. D., Gibson, J., Shirali, M., Coleman, J. R., Hagenaars, S. P., Ward, J., & Wigmore, E. M. J. N. n. (2019). Genome-wide meta-analysis of depression identifies 102 independent variants and highlights the importance of the prefrontal brain regions. *22*(3), 343-352.

Howard, D. M., Adams, M. J., Clarke, T. K., Hafferty, J. D., Gibson, J., Shirali, M., Coleman, J. R. I., Hagenaars, S. P., Ward, J., Wigmore, E. M., Alloza, C., Shen, X., Barbu, M. C., Xu, E. Y., Whalley, H. C., Marioni, R. E., Porteous, D. J., Davies, G., Deary, I. J., Hemani, G., Berger, K., Teismann, H., Rawal, R., Arolt, V., Baune, B. T., Dannlowski, U., Domschke, K., Tian, C., Hinds, D. A., andMe Research, T., Major Depressive Disorder Working Group of the Psychiatric Genomics, C., Trzaskowski, M., Byrne, E. M., Ripke, S., Smith, D. J., Sullivan, P. F., Wray, N. R., Breen, G., Lewis, C. M., & McIntosh, A. M. (2019). Genome-wide meta-analysis of depression identifies 102 independent variants and highlights the importance of the prefrontal brain regions. *Nat Neurosci*, *22*(3), 343-352. <https://doi.org/10.1038/s41593-018-0326-7>

Howard, D. M., Adams, M. J., Shirali, M., Clarke, T. K., Marioni, R. E., Davies, G., Coleman, J. R. I., Alloza, C., Shen, X., Barbu, M. C., Wigmore, E. M., Gibson, J., andMe Research, T., Hagenaars, S. P., Lewis, C. M., Ward, J., Smith, D. J., Sullivan, P. F., Haley, C. S., Breen, G., Deary, I. J., & McIntosh, A. M. (2018). Genome-wide association study of depression phenotypes in UK Biobank identifies variants in excitatory synaptic pathways. *Nat Commun*, *9*(1), 1470. <https://doi.org/10.1038/s41467-018-03819-3>

Hyde, C. L., Nagle, M. W., Tian, C., Chen, X., Paciga, S. A., Wendland, J. R., Tung, J. Y., Hinds, D. A., Perlis, R. H., & Winslow, A. R. (2016). Identification of 15 genetic loci associated with risk of major depression in individuals of European descent. *Nat Genet*, *48*(9), 1031-1036. <https://doi.org/10.1038/ng.3623>

Inouye, M., Kettunen, J., Soininen, P., Silander, K., Ripatti, S., Kumpula, L. S., Hamalainen, E., Jousilahti, P., Kangas, A. J., Mannisto, S., Savolainen, M. J., Jula, A., Leiviska, J., Palotie, A., Salomaa, V., Perola, M., Ala-Korpela, M., & Peltonen, L. (2010). Metabonomic, transcriptomic, and genomic variation of a population cohort. *Mol Syst Biol*, *6*, 441. <https://doi.org/10.1038/msb.2010.93>

Jarbin, H., Ivarsson, T., Andersson, M., Bergman, H., & Skarphedinsson, G. (2020). Screening efficiency of the Mood and Feelings Questionnaire (MFQ) and Short Mood and Feelings Questionnaire (SMFQ) in Swedish help seeking outpatients. *PLoS One*, *15*(3), e0230623. <https://doi.org/10.1371/journal.pone.0230623>

Mounier, N., & Kutalik, Z. J. b. (2021). Correction for sample overlap, winner’s curse and weak instrument bias in two-sample Mendelian Randomization.

Okbay, A., Baselmans, B. M., De Neve, J.-E., Turley, P., Nivard, M. G., Fontana, M. A., Meddens, S. F. W., Linnér, R. K., Rietveld, C. A., & Derringer, J. J. N. g. (2016). Genetic variants associated with subjective well-being, depressive symptoms, and neuroticism identified through genome-wide analyses. *48*(6), 624-633.

Perry, B. I., Upthegrove, R., Kappelmann, N., Jones, P. B., Burgess, S., & Khandaker, G. M. (2021). Associations of immunological proteins/traits with schizophrenia, major depression and bipolar disorder: A bi-directional two-sample mendelian randomization study. *Brain Behav Immun*, *97*, 176-185. <https://doi.org/10.1016/j.bbi.2021.07.009>

Sanderson, E., Glymour, M. M., Holmes, M. V., Kang, H., Morrison, J., Munafò, M. R., Palmer, T., Schooling, C. M., Wallace, C., & Zhao, Q. (2022). Mendelian randomization. *Nature Reviews Methods Primers*, *2*(1), 1-21.

Sanderson, E., Spiller, W., & Bowden, J. (2021). Testing and correcting for weak and pleiotropic instruments in two‐sample multivariable Mendelian randomization. *Statistics in medicine*, *40*(25), 5434-5452. <https://doi.org/10.1002/sim.9133>

Soininen, P., Kangas, A. J., Wurtz, P., Tukiainen, T., Tynkkynen, T., Laatikainen, R., Jarvelin, M. R., Kahonen, M., Lehtimaki, T., Viikari, J., Raitakari, O. T., Savolainen, M. J., & Ala-Korpela, M. (2009). High-throughput serum NMR metabonomics for cost-effective holistic studies on systemic metabolism. *Analyst*, *134*(9), 1781-1785. <https://doi.org/10.1039/b910205a>

Thabrew, H., Stasiak, K., Bavin, L. M., Frampton, C., & Merry, S. (2018). Validation of the Mood and Feelings Questionnaire (MFQ) and Short Mood and Feelings Questionnaire (SMFQ) in New Zealand help‐seeking adolescents. *International Journal of Methods in Psychiatric Research*, *27*(3), e1610. <https://doi.org/10.1002/mpr.1610>

Thapar, A., & McGuffin, P. (1998). Validity of the shortened Mood and Feelings Questionnaire in a community sample of children and adolescents: a preliminary research note. *Psychiatry Res*, *81*(2), 259-268. <https://doi.org/10.1016/s0165-1781(98)00073-0>

Turner, N., Joinson, C., Peters, T. J., Wiles, N., & Lewis, G. (2014). Validity of the Short Mood and Feelings Questionnaire in late adolescence. *Psychol Assess*, *26*(3), 752-762. <https://doi.org/10.1037/a0036572>

Wray, N. R., Ripke, S., Mattheisen, M., Trzaskowski, M., Byrne, E. M., Abdellaoui, A., Adams, M. J., Agerbo, E., Air, T. M., & Andlauer, T. M. J. N. g. (2018). Genome-wide association analyses identify 44 risk variants and refine the genetic architecture of major depression. *50*(5), 668-681.

Würtz, P., Kangas, A. J., Soininen, P., Lawlor, D. A., Davey Smith, G., & Ala-Korpela, M. (2017). Quantitative Serum Nuclear Magnetic Resonance Metabolomics in Large-Scale Epidemiology: A Primer on -Omic Technologies. *Am J Epidemiol*, *186*(9), 1084-1096. <https://doi.org/10.1093/aje/kwx016>

| **Supplementary Table 1: Main and auxiliary variables included to help inform the imputation** | | |
| --- | --- | --- |
| **Main Variables** |  |  |
| GlycA levels at 18y |  |  |
| GlycA levels at 24y |  |  |
| SMFQ score at 18y |  |  |
| SMFQ score at 24y |  |  |
| CIS-R diagnosis at age 18y |  |  |
| CIS-R diagnosis at age 24y |  |  |
| Age in months at 18y clinic |  |  |
| Age in months at 24y clinic |  |  |
| BMI at 18y clinic |  |  |
| BMI at 24y clinic |  |  |
| Ethnicity |  |  |
| Sex |  |  |
| Type of drinker at 24y clinic |  |  |
| Type of drinker at 18y clinic |  |  |
| Type of smoker at 24y clinic |  |  |
| Type of smoker at 18y clinic |  |  |
| Maternal highest education qualification |  |  |
| **Auxiliary Variables** |  |  |
| GlycA levels at age 7y |  |  |
| GlycA levels at age 15y |  |  |
| Moods and Feelings questionnaire at t8 |  |  |
| Moods and Feelings questionnaire at t7 |  |  |
| Moods and Feelings questionnaire at t6 |  |  |
| Moods and Feelings questionnaire at t4 |  |  |
| Moods and Feelings questionnaire at t3 |  |  |
| Moods and Feelings questionnaire at t9 |  |  |
| C-Reactive Protein levels at 9y clinic |  |  |
| C-Reactive Protein levels at 15y clinic |  |  |
| C-Reactive Protein levels at 24y clinic |  |  |
| Interleukin-6 levels at 9y clinic |  |  |
| Classic ACE score |  |  |
| Extended ACE score |  |  |
| Maternal social class |  |  |
| Maternal smoking status when pregnant |  |  |
| Household highest social class |  |  |

| **Supplementary Table 2: Harmonised SNPs for the association between GlycA and Depression with exposure information** | | | | | | | | | | |
| --- | --- | --- | --- | --- | --- | --- | --- | --- | --- | --- |
| **SNP** | **Effect Allele** | **Other Allele** | **Beta** | **Effect Allele Frequency** | **SE** | ***p*** | **R^2^** | **F-Stat** | **Steiger direction** | **Steiger *p*** |
| rs10455872 | G | A | -0.08 | 0.08 | 0.008 | 1.00E-25 | 0.001 | 104.14 | TRUE | 1.74E-20 |
| rs112875651 | A | G | -0.07 | 0.39 | 0.004 | 7.00E-65 | 0.002 | 277.81 | TRUE | 1.28E-49 |
| rs113354603 | A | G | 0.13 | 0.07 | 0.009 | 1.00E-50 | 0.002 | 245.53 | TRUE | 2.31E-40 |
| rs1168032 | G | A | 0.04 | 0.64 | 0.004 | 2.30E-20 | 0.001 | 77.20 | TRUE | 6.22E-15 |
| rs116843064 | A | G | -0.10 | 0.02 | 0.015 | 4.00E-11 | 0.000 | 43.71 | TRUE | 2.65E-08 |
| rs117155836 | A | G | 0.14 | 0.03 | 0.012 | 6.20E-30 | 0.001 | 122.58 | TRUE | 1.67E-20 |
| rs117733303 | G | A | -0.13 | 0.02 | 0.015 | 3.70E-17 | 0.001 | 68.59 | TRUE | 3.84E-14 |
| rs12032372 | C | T | -0.03 | 0.21 | 0.005 | 4.70E-09 | 0.0003 | 32.74 | TRUE | 3.11E-07 |
| rs12073837 | T | C | -0.03 | 0.28 | 0.005 | 1.60E-08 | 0.0003 | 30.43 | TRUE | 4.40E-06 |
| rs1260326 | C | T | -0.10 | 0.60 | 0.004 | 2.60E-125 | 0.005 | 559.31 | TRUE | 1.63E-99 |
| rs13108218 | G | A | -0.04 | 0.62 | 0.004 | 1.60E-19 | 0.001 | 79.25 | TRUE | 8.44E-16 |
| rs143578173 | T | C | -0.14 | 0.03 | 0.013 | 3.40E-27 | 0.001 | 123.79 | TRUE | 6.28E-21 |
| rs144018203 | C | G | 0.12 | 0.01 | 0.021 | 1.20E-08 | 0.0003 | 34.87 | TRUE | 2.92E-07 |
| rs146203232 | T | C | 0.05 | 0.07 | 0.008 | 5.30E-09 | 0.0003 | 33.97 | TRUE | 1.51E-07 |
| rs149807892 | T | C | -0.12 | 0.02 | 0.017 | 8.80E-14 | 0.0005 | 54.09 | TRUE | 7.79E-11 |
| rs150844304 | C | A | 0.07 | 0.03 | 0.013 | 4.40E-08 | 0.0003 | 29.74 | TRUE | 1.05E-06 |
| rs151083835 | G | C | 0.10 | 0.02 | 0.015 | 2.00E-12 | 0.0004 | 47.75 | TRUE | 4.60E-09 |
| rs17105232 | A | G | 0.03 | 0.25 | 0.005 | 1.40E-10 | 0.0003 | 39.59 | TRUE | 1.23E-08 |
| rs17580 | A | T | -0.05 | 0.05 | 0.010 | 8.80E-09 | 0.0002 | 27.97 | TRUE | 5.64E-05 |
| rs1801689 | C | A | -0.07 | 0.03 | 0.012 | 2.40E-10 | 0.0003 | 36.25 | TRUE | 4.09E-07 |
| rs182050989 | T | C | 0.07 | 0.03 | 0.012 | 1.50E-08 | 0.0003 | 33.54 | TRUE | 4.19E-07 |
| rs2070634 | G | T | 0.04 | 0.51 | 0.004 | 2.10E-19 | 0.001 | 81.11 | TRUE | 9.60E-14 |
| rs2294915 | T | C | -0.03 | 0.23 | 0.005 | 3.60E-11 | 0.0004 | 43.74 | TRUE | 2.34E-09 |
| rs2445818 | G | A | 0.05 | 0.93 | 0.008 | 2.60E-08 | 0.0003 | 29.48 | TRUE | 5.41E-06 |
| rs2497337 | T | C | 0.02 | 0.51 | 0.004 | 1.40E-08 | 0.0003 | 30.22 | TRUE | 1.40E-06 |
| rs28929474 | T | C | -0.28 | 0.02 | 0.015 | 3.80E-80 | 0.003 | 352.77 | TRUE | 1.62E-58 |
| rs325 | C | T | -0.09 | 0.10 | 0.007 | 5.60E-36 | 0.001 | 151.80 | TRUE | 1.93E-26 |
| rs4297946 | C | G | 0.02 | 0.47 | 0.004 | 1.50E-08 | 0.0003 | 30.26 | TRUE | 2.64E-04 |
| **SNP** | **Effect Allele** | **Other Allele** | **Beta** | **Effect Allele Frequency** | **SE** | ***p*** | **R^2^** | **F-Stat** | **Steiger direction** | **Steiger *p*** |
| rs4459081 | C | G | 0.03 | 0.65 | 0.004 | 3.70E-15 | 0.001 | 58.51 | TRUE | 4.38E-10 |
| rs55780214 | A | T | -0.03 | 0.29 | 0.005 | 4.40E-12 | 0.0004 | 45.10 | TRUE | 3.05E-09 |
| rs56188865 | C | T | -0.03 | 0.37 | 0.004 | 3.10E-11 | 0.0004 | 43.58 | TRUE | 4.57E-09 |
| rs58542926 | T | C | -0.06 | 0.07 | 0.008 | 7.80E-13 | 0.0004 | 49.62 | TRUE | 3.78E-08 |
| rs59296513 | A | G | -0.03 | 0.32 | 0.004 | 1.80E-09 | 0.0003 | 32.64 | TRUE | 3.71E-06 |
| rs59774409 | T | C | 0.08 | 0.08 | 0.007 | 3.30E-28 | 0.001 | 121.58 | TRUE | 5.00E-22 |
| rs62128087 | T | C | -0.04 | 0.16 | 0.006 | 3.30E-13 | 0.0005 | 53.68 | TRUE | 9.71E-10 |
| rs62259939 | A | G | 0.03 | 0.43 | 0.004 | 1.10E-09 | 0.0003 | 37.05 | TRUE | 1.38E-06 |
| rs62466318 | T | C | -0.09 | 0.20 | 0.005 | 8.50E-63 | 0.002 | 276.10 | TRUE | 1.05E-48 |
| rs6424109 | A | C | 0.04 | 0.87 | 0.006 | 1.40E-08 | 0.0003 | 32.22 | TRUE | 1.42E-06 |
| rs6452937 | G | A | -0.03 | 0.14 | 0.006 | 6.20E-09 | 0.0002 | 28.45 | TRUE | 1.68E-06 |
| rs6601299 | C | T | 0.07 | 0.90 | 0.007 | 2.90E-23 | 0.001 | 97.24 | TRUE | 1.44E-17 |
| rs6717858 | C | T | -0.02 | 0.40 | 0.004 | 3.80E-08 | 0.0002 | 25.98 | TRUE | 2.69E-05 |
| rs6734238 | G | A | 0.03 | 0.40 | 0.004 | 4.00E-09 | 0.0003 | 34.64 | TRUE | 1.04E-07 |
| rs676210 | A | G | -0.03 | 0.21 | 0.005 | 2.20E-08 | 0.0003 | 30.92 | TRUE | 3.07E-06 |
| rs687621 | G | A | 0.03 | 0.32 | 0.004 | 6.30E-11 | 0.0004 | 40.95 | TRUE | 2.00E-07 |
| rs72801474 | A | G | -0.04 | 0.09 | 0.007 | 1.10E-08 | 0.0003 | 32.91 | TRUE | 6.26E-07 |
| rs7697204 | T | C | 0.03 | 0.74 | 0.005 | 3.20E-11 | 0.0003 | 40.20 | TRUE | 3.08E-07 |
| rs77303550 | T | C | 0.16 | 0.19 | 0.005 | 1.00E-200 | 0.008 | 968.85 | TRUE | 3.90E-168 |
| rs78689694 | C | G | 0.03 | 0.13 | 0.006 | 4.70E-08 | 0.0003 | 31.18 | TRUE | 1.13E-06 |
| rs7924036 | T | G | -0.03 | 0.50 | 0.004 | 2.80E-12 | 0.0004 | 43.73 | TRUE | 2.12E-08 |
| rs79287178 | A | G | 0.10 | 0.03 | 0.012 | 7.70E-16 | 0.001 | 69.10 | TRUE | 4.10E-11 |
| rs964184 | C | G | -0.11 | 0.87 | 0.006 | 2.70E-68 | 0.003 | 295.06 | TRUE | 9.42E-53 |

| **Supplementary Table 3: Harmonised SNPs for the association between GlycA and depressive symptoms with exposure information** | | | | | | | | | | |
| --- | --- | --- | --- | --- | --- | --- | --- | --- | --- | --- |
| **SNP** | **Effect Allele** | **Other Allele** | **Beta** | **Effect Allele Frequency** | **SE** | ***p*** | **F-stat** | **R^2^** | **Steiger direction** | **Steiger *p*** |
| rs1021363 | G | A | -0.03 | 0.64 | 0.005 | 2.29E-11 | 206.66 | 8.88E-05 | TRUE | 0.84 |
| rs10235664 | C | T | -0.03 | 0.25 | 0.005 | 4.68E-08 | 137.83 | 6.07E-05 | TRUE | 0.52 |
| rs10913112 | T | C | -0.03 | 0.38 | 0.005 | 4.53E-09 | 161.51 | 6.78E-05 | TRUE | 0.24 |
| rs12919291 | C | G | 0.03 | 0.19 | 0.006 | 3.09E-09 | 163.62 | 7.07E-05 | TRUE | 0.39 |
| rs12967143 | C | G | -0.03 | 0.70 | 0.005 | 2.53E-13 | 249.60 | 0.000108 | TRUE | 0.00 |
| rs13037326 | T | C | 0.03 | 0.26 | 0.005 | 2.40E-10 | 184.90 | 8.00E-05 | TRUE | 0.06 |
| rs1367635 | C | T | 0.03 | 0.51 | 0.004 | 4.35E-09 | 160.00 | 6.92E-05 | TRUE | 0.02 |
| rs150186873 | C | A | 0.07 | 0.03 | 0.012 | 4.51E-09 | 156.88 | 6.88E-05 | TRUE | 0.01 |
| rs150346963 | T | C | 0.03 | 0.41 | 0.004 | 1.16E-10 | 194.14 | 8.27E-05 | TRUE | 0.01 |
| rs17641524 | T | C | -0.03 | 0.21 | 0.005 | 1.50E-08 | 149.47 | 6.41E-05 | TRUE | 0.03 |
| rs1931388 | G | A | -0.03 | 0.40 | 0.004 | 1.68E-11 | 209.75 | 8.99E-05 | TRUE | 0.33 |
| rs1950829 | G | A | -0.03 | 0.52 | 0.004 | 4.74E-12 | 220.44 | 9.54E-05 | TRUE | 0.00 |
| rs198457 | T | C | -0.03 | 0.19 | 0.006 | 1.90E-08 | 151.95 | 6.33E-05 | TRUE | 0.35 |
| rs2111592 | A | G | 0.03 | 0.31 | 0.005 | 1.35E-08 | 149.12 | 6.53E-05 | TRUE | 0.03 |
| rs2214123 | G | A | -0.03 | 0.65 | 0.005 | 8.56E-09 | 155.77 | 6.72E-05 | TRUE | 0.06 |
| rs2232423 | G | A | -0.06 | 0.11 | 0.007 | 1.14E-18 | 363.47 | 0.000157 | TRUE | 0.35 |
| rs2418449 | C | T | -0.03 | 0.28 | 0.005 | 4.25E-09 | 159.65 | 6.85E-05 | TRUE | 0.38 |
| rs247910 | G | A | 0.02 | 0.46 | 0.004 | 4.71E-08 | 139.48 | 6.07E-05 | TRUE | 0.14 |
| rs2522831 | C | T | 0.02 | 0.47 | 0.004 | 2.11E-08 | 143.71 | 6.23E-05 | TRUE | 0.03 |
| rs2568958 | A | G | 0.04 | 0.60 | 0.004 | 2.90E-18 | 349.35 | 0.000151 | TRUE | 0.00 |
| rs28541419 | G | C | -0.03 | 0.23 | 0.005 | 1.76E-08 | 151.48 | 6.30E-05 | TRUE | 0.31 |
| rs2876520 | G | C | 0.03 | 0.47 | 0.004 | 2.24E-09 | 168.47 | 7.31E-05 | TRUE | 0.01 |
| rs30266 | A | G | 0.04 | 0.33 | 0.005 | 1.43E-15 | 295.13 | 0.000127 | TRUE | 0.04 |
| rs354155 | C | G | -0.04 | 0.09 | 0.008 | 1.75E-09 | 169.03 | 7.16E-05 | TRUE | 0.06 |
| rs3807865 | A | G | 0.03 | 0.41 | 0.004 | 1.09E-12 | 232.75 | 9.92E-05 | TRUE | 0.08 |
| rs4141983 | C | T | -0.03 | 0.33 | 0.005 | 9.69E-09 | 153.25 | 6.58E-05 | TRUE | 0.03 |
| rs4497414 | C | T | 0.03 | 0.44 | 0.004 | 2.93E-11 | 208.82 | 8.74E-05 | TRUE | 0.12 |
| rs4730387 | A | T | 0.02 | 0.47 | 0.004 | 4.12E-08 | 141.05 | 6.12E-05 | TRUE | 0.18 |
| **SNP** | **Effect Allele** | **Other Allele** | **Beta** | **Effect Allele Frequency** | **SE** | ***p*** | **F-stat** | **R^2^** | **Steiger direction** | **Steiger *p*** |
| rs4799949 | T | C | -0.03 | 0.67 | 0.005 | 1.40E-10 | 189.13 | 8.06E-05 | TRUE | 0.38 |
| rs4936276 | C | G | 0.03 | 0.62 | 0.004 | 3.57E-10 | 181.84 | 7.98E-05 | TRUE | 0.47 |
| rs508502 | T | C | -0.03 | 0.30 | 0.005 | 3.56E-08 | 146.24 | 6.05E-05 | TRUE | 0.03 |
| rs59082935 | T | C | 0.04 | 0.13 | 0.007 | 3.07E-08 | 153.21 | 6.05E-05 | TRUE | 0.02 |
| rs59283172 | A | G | -0.04 | 0.11 | 0.007 | 2.41E-08 | 146.75 | 6.21E-05 | TRUE | 0.02 |
| rs61914045 | A | G | 0.03 | 0.20 | 0.005 | 7.96E-09 | 154.82 | 6.55E-05 | TRUE | 0.29 |
| rs62535714 | A | G | 0.03 | 0.16 | 0.006 | 4.69E-09 | 157.60 | 6.83E-05 | TRUE | 0.20 |
| rs66511648 | C | T | 0.03 | 0.28 | 0.005 | 6.03E-10 | 179.50 | 7.65E-05 | TRUE | 0.21 |
| rs7152906 | C | T | 0.03 | 0.52 | 0.004 | 1.87E-09 | 166.28 | 7.20E-05 | TRUE | 0.08 |
| rs7241572 | A | G | 0.03 | 0.20 | 0.005 | 2.43E-09 | 169.97 | 7.15E-05 | TRUE | 0.72 |
| rs72948506 | A | G | 0.03 | 0.30 | 0.005 | 1.71E-08 | 146.87 | 6.36E-05 | TRUE | 0.69 |
| rs7538938 | C | T | 0.03 | 0.56 | 0.004 | 7.29E-09 | 155.35 | 6.81E-05 | TRUE | 0.31 |
| rs754287 | A | T | -0.03 | 0.37 | 0.005 | 1.31E-10 | 194.05 | 8.25E-05 | TRUE | 0.18 |
| rs7551758 | G | T | 0.03 | 0.53 | 0.004 | 5.11E-11 | 199.51 | 8.66E-05 | TRUE | 0.02 |
| rs76954012 | A | T | 0.04 | 0.09 | 0.007 | 2.41E-08 | 143.42 | 6.20E-05 | TRUE | 0.49 |
| rs7725715 | A | G | 0.03 | 0.53 | 0.004 | 1.61E-11 | 209.43 | 9.09E-05 | TRUE | 0.61 |
| rs843812 | A | G | 0.02 | 0.41 | 0.004 | 1.41E-08 | 149.07 | 6.35E-05 | TRUE | 0.03 |
| rs9364755 | G | A | 0.03 | 0.23 | 0.005 | 3.49E-08 | 140.28 | 6.16E-05 | TRUE | 0.72 |
| rs9529218 | T | C | -0.03 | 0.20 | 0.005 | 2.23E-10 | 187.24 | 7.92E-05 | TRUE | 0.36 |
| rs9536381 | T | C | 0.03 | 0.33 | 0.005 | 2.62E-08 | 142.95 | 6.14E-05 | TRUE | 0.26 |
| rs9831648 | T | G | -0.03 | 0.77 | 0.005 | 1.59E-08 | 149.30 | 6.30E-05 | TRUE | 0.33 |

| **Supplementary Table 4: Harmonised SNPs for the association between Depression and GlycA with exposure information** | | | | | | | | | | |
| --- | --- | --- | --- | --- | --- | --- | --- | --- | --- | --- |
| **SNP** | **Effect Allele** | **Other Allele** | **Beta** | **Effect Allele Frequency** | **SE** | ***p*** | **F-stat** | **R^2^** | **Steiger direction** | **Steiger *p*** |
| rs1021363 | G | A | -0.03 | 0.64 | 0.005 | 2.29E-11 | 206.66 | 8.88E-05 | TRUE | 0.84 |
| rs10235664 | C | T | -0.03 | 0.25 | 0.005 | 4.68E-08 | 137.83 | 6.07E-05 | TRUE | 0.52 |
| rs10913112 | T | C | -0.03 | 0.38 | 0.005 | 4.53E-09 | 161.51 | 6.78E-05 | TRUE | 0.24 |
| rs12919291 | C | G | 0.03 | 0.19 | 0.006 | 3.09E-09 | 163.62 | 7.07E-05 | TRUE | 0.39 |
| rs12967143 | C | G | -0.03 | 0.70 | 0.005 | 2.53E-13 | 249.60 | 0.000108 | TRUE | 0.00 |
| rs13037326 | T | C | 0.03 | 0.26 | 0.005 | 2.40E-10 | 184.90 | 8.00E-05 | TRUE | 0.06 |
| rs1367635 | C | T | 0.03 | 0.51 | 0.004 | 4.35E-09 | 160.00 | 6.92E-05 | TRUE | 0.02 |
| rs150186873 | C | A | 0.07 | 0.03 | 0.012 | 4.51E-09 | 156.88 | 6.88E-05 | TRUE | 0.01 |
| rs150346963 | T | C | 0.03 | 0.41 | 0.004 | 1.16E-10 | 194.14 | 8.27E-05 | TRUE | 0.01 |
| rs17641524 | T | C | -0.03 | 0.21 | 0.005 | 1.50E-08 | 149.47 | 6.41E-05 | TRUE | 0.03 |
| rs1931388 | G | A | -0.03 | 0.40 | 0.004 | 1.68E-11 | 209.75 | 8.99E-05 | TRUE | 0.33 |
| rs1950829 | G | A | -0.03 | 0.52 | 0.004 | 4.74E-12 | 220.44 | 9.54E-05 | TRUE | 0.00 |
| rs198457 | T | C | -0.03 | 0.19 | 0.006 | 1.90E-08 | 151.95 | 6.33E-05 | TRUE | 0.35 |
| rs2111592 | A | G | 0.03 | 0.31 | 0.005 | 1.35E-08 | 149.12 | 6.53E-05 | TRUE | 0.03 |
| rs2214123 | G | A | -0.03 | 0.65 | 0.005 | 8.56E-09 | 155.77 | 6.72E-05 | TRUE | 0.06 |
| rs2232423 | G | A | -0.06 | 0.11 | 0.007 | 1.14E-18 | 363.47 | 0.000157 | TRUE | 0.35 |
| rs2418449 | C | T | -0.03 | 0.28 | 0.005 | 4.25E-09 | 159.65 | 6.85E-05 | TRUE | 0.38 |
| rs247910 | G | A | 0.02 | 0.46 | 0.004 | 4.71E-08 | 139.48 | 6.07E-05 | TRUE | 0.14 |
| rs2522831 | C | T | 0.02 | 0.47 | 0.004 | 2.11E-08 | 143.71 | 6.23E-05 | TRUE | 0.03 |
| rs2568958 | A | G | 0.04 | 0.60 | 0.004 | 2.90E-18 | 349.35 | 0.000151 | TRUE | 0.00 |
| rs28541419 | G | C | -0.03 | 0.23 | 0.005 | 1.76E-08 | 151.48 | 6.30E-05 | TRUE | 0.31 |
| rs30266 | A | G | 0.04 | 0.33 | 0.005 | 1.43E-15 | 295.13 | 0.000127 | TRUE | 0.04 |
| rs354155 | C | G | -0.04 | 0.09 | 0.008 | 1.75E-09 | 169.03 | 7.16E-05 | TRUE | 0.06 |
| rs3807865 | A | G | 0.03 | 0.41 | 0.004 | 1.09E-12 | 232.75 | 9.92E-05 | TRUE | 0.08 |
| rs4141983 | C | T | -0.03 | 0.33 | 0.005 | 9.69E-09 | 153.25 | 6.58E-05 | TRUE | 0.03 |
| rs4497414 | C | T | 0.03 | 0.44 | 0.004 | 2.93E-11 | 208.82 | 8.74E-05 | TRUE | 0.12 |
| rs4799949 | T | C | -0.03 | 0.67 | 0.005 | 1.40E-10 | 189.13 | 8.06E-05 | TRUE | 0.38 |
| **SNP** | **Effect Allele** | **Other Allele** | **Beta** | **Effect Allele Frequency** | **SE** | ***p*** | **F-stat** | **R^2^** | **Steiger direction** | **Steiger *p*** |
| rs4936276 | C | G | 0.03 | 0.62 | 0.004 | 3.57E-10 | 181.84 | 7.98E-05 | TRUE | 0.47 |
| rs508502 | T | C | -0.03 | 0.30 | 0.005 | 3.56E-08 | 146.24 | 6.05E-05 | TRUE | 0.03 |
| rs59082935 | T | C | 0.04 | 0.13 | 0.007 | 3.07E-08 | 153.21 | 6.05E-05 | TRUE | 0.02 |
| rs59283172 | A | G | -0.04 | 0.11 | 0.007 | 2.41E-08 | 146.75 | 6.21E-05 | TRUE | 0.02 |
| rs61914045 | A | G | 0.03 | 0.20 | 0.005 | 7.96E-09 | 154.82 | 6.55E-05 | TRUE | 0.29 |
| rs62535714 | A | G | 0.03 | 0.16 | 0.006 | 4.69E-09 | 157.60 | 6.83E-05 | TRUE | 0.20 |
| rs66511648 | C | T | 0.03 | 0.28 | 0.005 | 6.03E-10 | 179.50 | 7.65E-05 | TRUE | 0.21 |
| rs7152906 | C | T | 0.03 | 0.52 | 0.004 | 1.87E-09 | 166.28 | 7.20E-05 | TRUE | 0.08 |
| rs7241572 | A | G | 0.03 | 0.20 | 0.005 | 2.43E-09 | 169.97 | 7.15E-05 | TRUE | 0.72 |
| rs72948506 | A | G | 0.03 | 0.30 | 0.005 | 1.71E-08 | 146.87 | 6.36E-05 | TRUE | 0.69 |
| rs7538938 | C | T | 0.03 | 0.56 | 0.004 | 7.29E-09 | 155.35 | 6.81E-05 | TRUE | 0.31 |
| rs754287 | A | T | -0.03 | 0.37 | 0.005 | 1.31E-10 | 194.05 | 8.25E-05 | TRUE | 0.18 |
| rs7551758 | G | T | 0.03 | 0.53 | 0.004 | 5.11E-11 | 199.51 | 8.66E-05 | TRUE | 0.02 |
| rs76954012 | A | T | 0.04 | 0.09 | 0.007 | 2.41E-08 | 143.42 | 6.20E-05 | TRUE | 0.49 |
| rs7725715 | A | G | 0.03 | 0.53 | 0.004 | 1.61E-11 | 209.43 | 9.09E-05 | TRUE | 0.61 |
| rs843812 | A | G | 0.02 | 0.41 | 0.004 | 1.41E-08 | 149.07 | 6.35E-05 | TRUE | 0.03 |
| rs9364755 | G | A | 0.03 | 0.23 | 0.005 | 3.49E-08 | 140.28 | 6.16E-05 | TRUE | 0.72 |
| rs9529218 | T | C | -0.03 | 0.20 | 0.005 | 2.23E-10 | 187.24 | 7.92E-05 | TRUE | 0.36 |
| rs9536381 | T | C | 0.03 | 0.33 | 0.005 | 2.62E-08 | 142.95 | 6.14E-05 | TRUE | 0.26 |
| rs9831648 | T | G | -0.03 | 0.77 | 0.005 | 1.59E-08 | 149.30 | 6.30E-05 | TRUE | 0.33 |

| **Supplementary Table 5: Description of MR, MR methods and sensitivity analyses** | | |
| --- | --- | --- |
| Method | Description | |
| Main analysis | | |
| Mendelian Randomisation (MR) | MR is an instrumental variable approach , using genetic variants as instruments for a modifiable exposure. It interrogates the causal effect of an exposure on an outcome, by utilising the random assortment of genetic variants (during gamete formation) from parents to offspring and as a result, the potential bias from confounding and reverse causation are minimised. MR is bound by three assumptions: (i) the genetic variants are statistically strongly associated with the exposure of interest and relevant to the population to which inference is being made (the relevance assumption); (ii) there is no confounding of the SNP-outcome association; and (iii) any effect of the genetic instrument on the outcome is only via the exposure (Sanderson et al., 2022) (supplementary figure 4). | |
| Inverse Variance Weighted (IVW) | A Wald ratio estimate is calculated for each genetic variant and summarised using the weighted regression of the SNP-exposure estimates on SNP-outcome estimates, where the intercept was constrained to zero. IVW assumes that there is no correlation between the association of the SNP exposure and SNP-pleiotropic path (the Instrument Strength Independent of Direct Effect (InSIDE) assumption) in the presence of horizontal pleiotropic paths. | |
| Methods to explore MR assumption | | |
| F-Statistic | Relevance assumption and weak instrument bias | Investigating the strength of genetic instruments using the F-statistics and proportion explained (R^2^) by each SNP. F-statistics >10 indicate that the estimates are not substantially biased by weak instruments (Davies et al., 2018) |
| MR-Egger | Horizontal pleiotropy. | Unlike the IVW, MR-Egger does not constrain the regression line to go through zero. Therefore, the MR-Egger regression represents the estimate of the causal effect controlling for unbalanced horizontal pleiotropy. A non-null MR-Egger intercept provides evidence of horizontal pleiotropy. MR-Egger has less statistical power than IVW and there should be no violation of the InSIDE assumption |
| Weighted Median | Horizontal pleiotropy | Provides an unbiased estimate when up to 50% of the SNPs used in the instrument violate the IV assumption |
| Weighted Mode | Horizontal pleiotropy | Uses the mode of the IVW empirical density function as the effect estimate |
| Cochran’s Q / Rucker’s Q statistic | Horizontal pleiotropy | Test for between-SNP heterogeneity in the IVW and MR-Egger analysis. |
| MR-Egger intercept | Horizontal pleiotropy | Under the assumption that pleiotropy is independent of the SNP-exposure association will give evidence for pleiotropic effects. |
| Sample definition/ underlying population | Confounding (via population stratification) | To mitigate bias caused by population stratification, which can confound the genetic instrument-outcome association and therefore violate the independence assumption, we restricted analyses to GWAS’ of the same underlying population (European ancestry participants only). |
| Radial MR | Horizontal pleiotropy | The method is similar to IVW but using a simulation-based approach fits a radial IVW model and provides an effect estimate, allowing outliers to be identified using Cochran’s Q statistic. The same can be done for a radial MR-Egger model. |
| Steiger filtering | Directionality of effects | Determines whether the proportion of variance explained by each SNP was larger in the exposure than that in the outcome. SNPs that did not pass Steiger filtering were excluded (Hemani et al., 2017). |
| MR Pleiotropy Residual Sum and Outlier global test (MR-PRESSO) | Horizontal pleiotropy | This method evaluates horizontal pleiotropy and corrects for it via outlier removal. It then tests for significant distortion in the causal estimate before and after outlier removal. |
| MR-Lap | Sample overlap, Winners Curse and weak instrument bias | MR-Lap uses cross-trait LD-score regression (LDSC) to simultaneously account and correct for winner’s curse, weak instrument bias and sample overlap (Mounier & Kutalik, 2021). |

| **Supplementary table 6: Statistical Power in the bidirectional Mendelian randomization analyses of Inflammation and depression diagnosis/symptoms.** | | | | | |  |
| --- | --- | --- | --- | --- | --- | --- |
| Exposure | Outcome | Sample size | Cases | Mean difference per unit change in exposure/ odds ratio | Power | |
| GlycA | Depressive symptoms | 161,460 | N/A | 0.011 | 0.05 | |
| GlycA | Depression | 807,553 | 170,756 | 1.02 | 0.44 | |
| Depression | GlycA | 115,078 | N/A | 0.02 | 0.85 | |
| NB: α = 0.05 |  |  |  |  |  | |

| **Supplementary Table 7: The distributions of observed and imputed characteristics at ages 18y and 24y** | | | | | |
| --- | --- | --- | --- | --- | --- |
|  | **Observed** | | |  | **Imputed (N=4021)** |
| **Variables** | **Categories** | **N** | **Mean (SE) for continuous variables Number (%) for categorical variables** | **% data imputed** | **Mean (SE) for continuous variables Number (%) for categorical variables** |
| Ethnicity | White | 3562 | 98.05 | 9.65 | 97.94 |
|  | Non-White | 71 | 1.95 |  | 2.06 |
| Sex | Male | 1696 | 42.18 | 0 | 42.18 |
|  | Female | 2325 | 57.82 |  | 57.82 |
| BMI at 18y clinic (kg/m^2^) |  | 3537 | 22.73 (0.07) | 12.04 | 22.74 (0.06) |
| BMI at 24y clinic (kg/m2) |  | 2996 | 24.74 (0.09) | 25.49 | 24.90 (0.09) |
| Age at 18y clinic in months |  | 3609 | 213.41 (0.08) | 10.25 | 213.38 (0.08) |
| Age at 24y clinic in months |  | 3025 | 293.17 (0.17) | 24.77 | 293.28 (0.19) |
| SMFQ score at 18y |  | 3392 | 6.45 (0.09) | 15.64 | 6.53 (0.09) |
| SMFQ score at 24y |  | 2718 | 6.84 (0.11) | 32.40 | 6.88 (0.11) |
| CIS-R at 18y | No depression diagnosis | 3052 | 92.54 | 17.98 | 92.17 |
|  | Diagnosis of depression | 247 | 7.46 |  | 7.83 |
| CIS-R at 24y | No diagnosis of depression | 2690 | 89.76 | 25.47 | 89.62 |
|  | Depression diagnosis | 307 | 10.24 |  | 10.38 |
| Average GlycA at age 18y (mmol/L) |  | 2918 | 1.22 (0.002) | 27.43 | 1.22 (0.002) |
| Average GlycA at age 24y (mmol/L) |  | 2836 | 1.23 (0.003) | 29.47 | 1.23 (0.003) |
| Maternal smoking during pregnancy | Never | 3104 | 84.44 | 8.58 | 84.28 |
|  | Temporary | 147 | 4.00 |  | 4.03 |
|  | Throughout | 425 | 11.56 |  | 11.69 |
|  | **Observed** |  |  |  | **Imputed (N=4021)** |
| **Variables** | **Categories** | **N** | **Mean (SE) for continuous variables Number (%) for categorical variables** | **% data imputed** | **Mean (SE) for continuous variables Number (%) for categorical variables** |
| Maternal self-reported highest education qualification | Below O-level | 536 | 15.12 | 11.81 | 15.59 |
|  | O-Level/Vocational | 1221 | 34.43 |  | 34.40 |
|  | A-Level | 1051 | 29.64 |  | 29.51 |
|  | Degree or above | 738 | 20.81 |  | 20.49 |
| Smoking Status at 18y | Non-smoker | 1551 | 65.47 | 41.08 | 65.37 |
|  | Infrequent Smoker | 341 | 14.39 |  | 15.18 |
|  | Frequent Smoker | 477 | 20.14 |  | 19.45 |
| Smoking Status at 24y | Non-smoker | 1109 | 37.10 | 25.67 | 36.49 |
|  | Infrequent Smoker | 1382 | 46.24 |  | 46.21 |
|  | Frequent Smoker | 498 | 16.66 |  | 17.30 |
| Drinking Status at 18y | Non-Drinker | 544 | 15.77 | 14.20 | 16.06 |
|  | Infrequent Drinker | 1465 | 42.46 |  | 42.74 |
|  | Frequent Drinker | 1441 | 41.77 |  | 41.19 |
| Drinking Status at 24y | Non-Drinker | 50 | 2.22 | 43.87 | 2.41 |
|  | Infrequent Drinker | 1583 | 70.18 |  | 70.49 |
|  | Frequent Drinker | 623 | 27.60 |  | 27.10 |
| Household social economic position | Non-manual | 2915 | 86.55 | 16.24 | 85.95 |
|  | Manual | 453 | 13.45 |  | 14.05 |

| **Supplementary Table 8: Bidirectional associations between continuous SMFQ and GlycA cross-sectionally at ages 18y and 24y using complete case analysis** | | | | | | | |
| --- | --- | --- | --- | --- | --- | --- | --- |
|  |  | **Model 1** | |  | **Model 2*** | |  |
|  | **N** | **Mean difference per SD increase in exposure (SE)** | **95% CI** | ***p*** | **Mean difference per SD increase in exposure (SE)** | **95% CI** | ***p*** |
| GlycA at 18y in relation to SMFQ score at 24y | 575 | 0.05 (0.04) | -0.03, 0.13 | 0.24 | 0.01 (0.04) | -0.08, 0.09 | 0.90 |
| SMFQ score at 18y in relation to GlycA levels at 24y | 575 | 0.04 (0.04) | -0.05, 0.12 | 0.37 | 0.01 (0.04) | -0.07, 0.09 | 0.87 |
|  | **N** | **OR** | **95% CI** | ***p*** | **OR** | **95% CI** | ***p*** |
| GlycA at 18y in relation to depressive episode at 24y | 575 | 1.28 (0.19) | 0.96, 1.70 | 0.10 | 1.18 (0.20) | 0.86, 1.64 | 0.31 |
| Depressive episode at 18y in relation to GlycA at 24y | **N** | **Mean difference per SD increase in exposure** | **95% CI** |  | **Mean difference per SD increase in exposure** | **95% CI** |  |
| Depression (as measured by CIS-R) at 18y on GlycA at 24y | 575 | 0.11 (0.15) | -0.19, 0.41 | 0.47 | 0.03 (0.15) | -0.26, 0.31 | 0.86 |
| * Adjusted for smoking status, drinking status, age in months at baseline, sex, ethnicity, maternal highest education qualification and BMI at 18y. | | | | | | |  |

| **Supplementary Table 9*:* Genetic instruments and sample sizes used to estimate SNP-exposure and SNP-outcome associations** | | | | | |
| --- | --- | --- | --- | --- | --- |
| **GWAS** | **Instrument** | **Outcome** | **Number of SNPs** | **Instrument F-statistics ^a^** | **Exposure**  **Sample Size** |
| Burges | GlycA | Depression | 51 | Min = 25.98  Median = 43.73  Max = 968.85 | 115,078 |
| Burges | GlycA | Depressive symptoms | 42 | Min=25.98  Median = 43.65  Max=968.85 | 115,078 |
| Howard *et al.* (2018)(D. M. Howard et al., 2019) | Depression | GlycA | 47 | Min = 137.83  Median = 160.00  Max =363.47 | 500,199 |
| Okbay *et al.* (2016) (Okbay et al., 2016) | Depressive Symptoms | GlycA | 2 | Min = 47.83  Median = 49.07  Max =50.31 | 161,460 |
| Ahluwalia et al.  (2021) (Ahluwalia et al., 2021) | IL-6 | Depression | 1 | 2.71 | 3301 |
| Howard *et al.* (2018)(D. M. Howard et al., 2019) | Depression | IL-6 | 20 | Min = 164.98  Median= 139.48 Max=220.44 | 500,199 |
| ^a^Instrument strength F-statistics are based on the formulae $R^{2}=2*MAF*\left( 1-MAF \right)*{beta}^{2}$, where MAF=Minor allele frequency, and $F= \frac{R^{2}* ( N-2 )}{1-R^{2}}$ ; as described in Shim *et al.*^17^ and Palmer *et al.*^18^ previously.  These results are post-steiger filtering. | | | | | |

| \| \| **Supplementary Table 10: Harmonised SNPs for the association between IL-6 and Depression with exposure information** \| \| --- \| \| \| --- \| --- \| | | | | | | | | | | |
| --- | --- | --- | --- | --- | --- | --- | --- | --- | --- | --- | --- | --- |
| **SNP** | **Effect Allele** | **Other Allele** | **Beta** | **Effect Allele Frequency** | **SE** | ***p*** | **F-stat** | **R^2^** | **Steiger direction** | **Steiger *p*** |
| rs6684439 | T | C | 0.06 | 0.15 | 0.01 | 0.99 | 2.708 | 0.001 | False | 0.935 |

| **Supplementary Table 11: Harmonised SNPs for the association between Depression and IL6 with exposure information** | | | | | | | | | | |
| --- | --- | --- | --- | --- | --- | --- | --- | --- | --- | --- |
| **SNP** | **Effect Allele** | **Other Allele** | **Beta** | **Effect Allele Frequency** | **SE** | ***p*** | **R^2^** | **F-stat** | **Steiger direction** | **Steiger *p*** |
| rs1021363 | G | A | -0.03 | 0.6434 | 0.0045 | 2.29E-11 | 0.000413 | 206.6595 | TRUE | 0.651115 |
| rs10913112 | T | C | -0.0262 | 0.378 | 0.0045 | 4.53E-09 | 0.000323 | 161.5087 | TRUE | 0.662549 |
| rs12967143 | C | G | -0.0345 | 0.7012 | 0.0047 | 2.53E-13 | 0.000499 | 249.6022 | TRUE | 0.7218 |
| rs13037326 | T | C | 0.031 | 0.2597 | 0.0049 | 2.40E-10 | 0.00037 | 184.899 | FALSE | 0.552214 |
| rs1931388 | G | A | -0.0295 | 0.4042 | 0.0044 | 1.68E-11 | 0.000419 | 209.7461 | FALSE | 0.355174 |
| rs1950829 | G | A | -0.0297 | 0.5173 | 0.0043 | 4.74E-12 | 0.000441 | 220.4424 | FALSE | 0.721695 |
| rs2111592 | A | G | 0.0263 | 0.3141 | 0.0046 | 1.35E-08 | 0.000298 | 149.1217 | TRUE | 0.823155 |
| rs2214123 | G | A | -0.0261 | 0.6466 | 0.0045 | 8.56E-09 | 0.000311 | 155.7721 | TRUE | 0.662857 |
| rs2232423 | G | A | -0.062 | 0.1056 | 0.007 | 1.14E-18 | 0.000726 | 363.4675 | FALSE | 0.203539 |
| rs2418449 | C | T | -0.0281 | 0.281 | 0.0048 | 4.25E-09 | 0.000319 | 159.6458 | TRUE | 0.955458 |
| rs2522831 | C | T | 0.024 | 0.4739 | 0.0043 | 2.11E-08 | 0.000287 | 143.7055 | TRUE | 0.720365 |
| rs2568958 | A | G | 0.0382 | 0.6042 | 0.0044 | 2.90E-18 | 0.000698 | 349.3474 | TRUE | 0.911139 |
| rs2876520 | G | C | 0.026 | 0.4688 | 0.0043 | 2.24E-09 | 0.000337 | 168.465 | TRUE | 0.72582 |
| rs354155 | C | G | -0.0449 | 0.0923 | 0.0075 | 1.75E-09 | 0.000338 | 169.0264 | TRUE | 0.885307 |
| rs3807865 | A | G | 0.031 | 0.4105 | 0.0044 | 1.09E-12 | 0.000465 | 232.752 | FALSE | 0.461647 |
| rs4141983 | C | T | -0.0264 | 0.326 | 0.0046 | 9.69E-09 | 0.000306 | 153.2461 | TRUE | 0.969607 |
| rs4936276 | C | G | 0.0278 | 0.622 | 0.0044 | 3.57E-10 | 0.000363 | 181.8447 | FALSE | 0.832895 |
| rs7152906 | C | T | 0.0258 | 0.5196 | 0.0043 | 1.87E-09 | 0.000332 | 166.275 | TRUE | 0.709202 |
| rs7241572 | A | G | 0.0323 | 0.2047 | 0.0054 | 2.43E-09 | 0.00034 | 169.9701 | FALSE | 0.585933 |
| rs7551758 | G | T | 0.0283 | 0.5329 | 0.0043 | 5.11E-11 | 0.000399 | 199.5137 | FALSE | 0.720536 |
| rs843812 | A | G | 0.0248 | 0.4117 | 0.0044 | 1.41E-08 | 0.000298 | 149.0677 | FALSE | 0.390123 |
| rs9364755 | G | A | 0.0283 | 0.2262 | 0.0051 | 3.49E-08 | 0.00028 | 140.2772 | FALSE | 0.398088 |
| rs9536381 | T | C | 0.0255 | 0.3259 | 0.0046 | 2.62E-08 | 0.000286 | 142.95 | FALSE | 0.571334 |


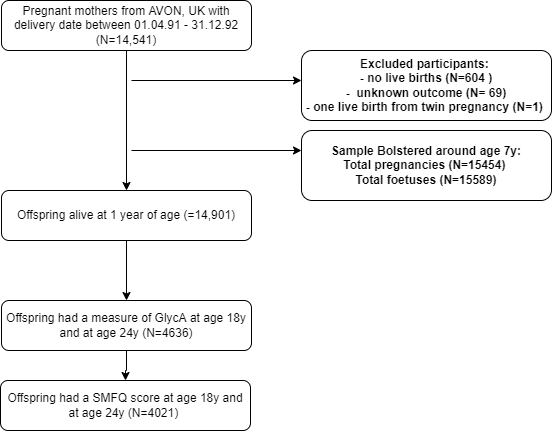


Supplementary Figure 1: Flowchart of included ALSPAC participants


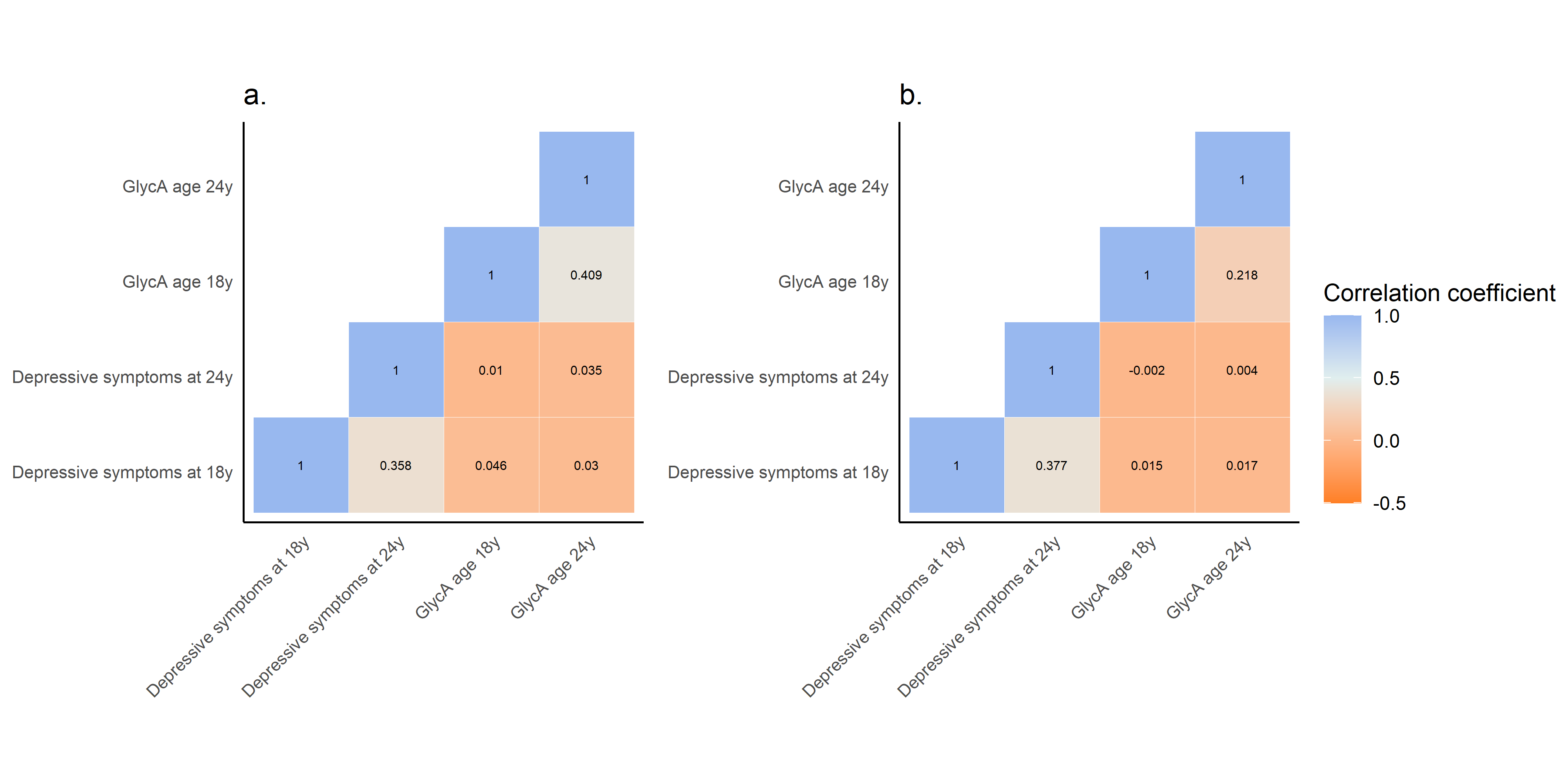


Supplementary Figure 2: Unadjusted (plot a) and adjusted (plot b) correlation matrices of the primary exposure and outcome measures.

*NB. Adjusted for smoking and drinking status at age 18 and 24, sex, ethnicity, SEP and age and BMI at baseline.*

Supplementary Figure 3: Bidirectional relationship between genetically predicted IL-6 on MDD


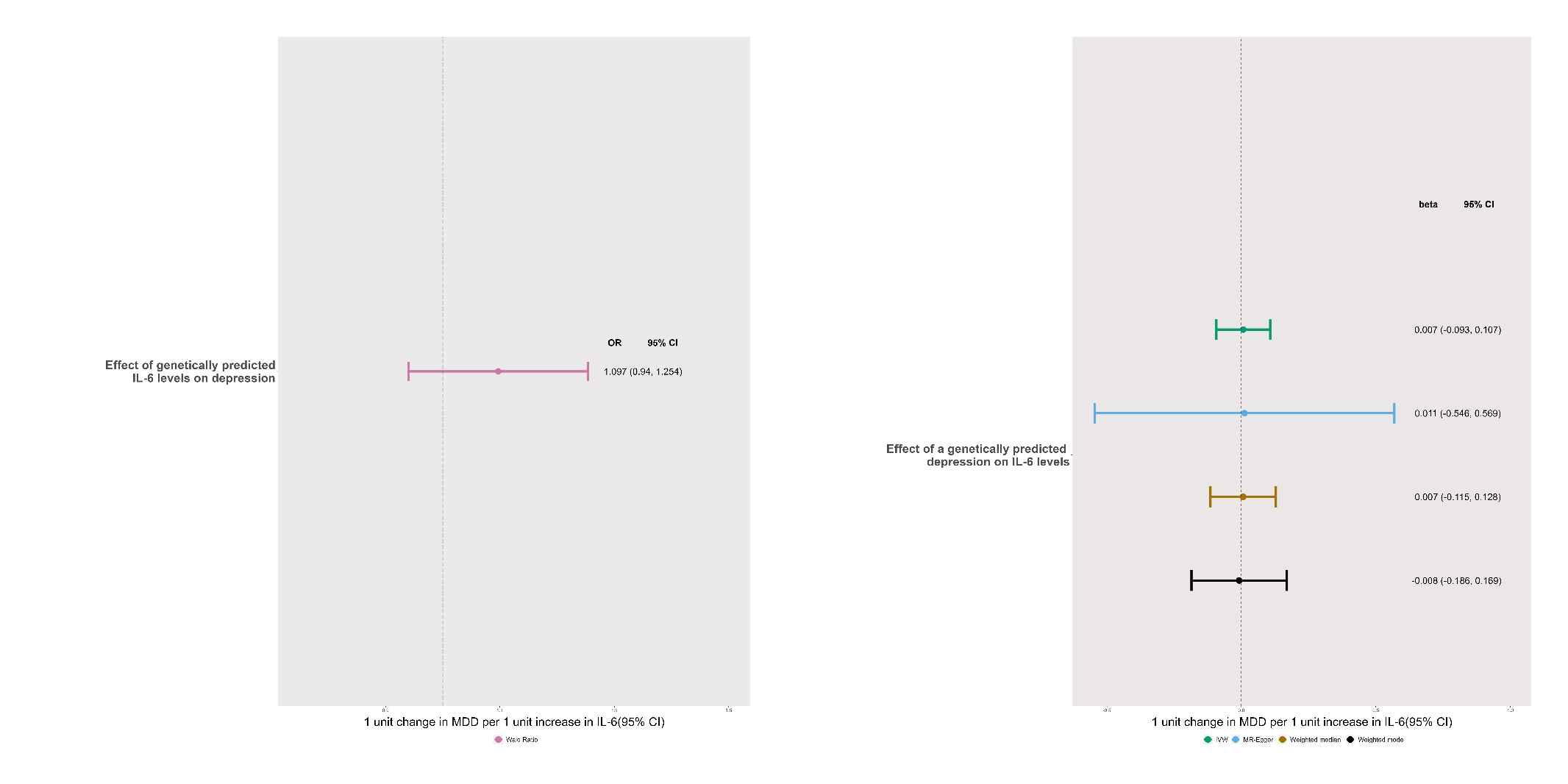

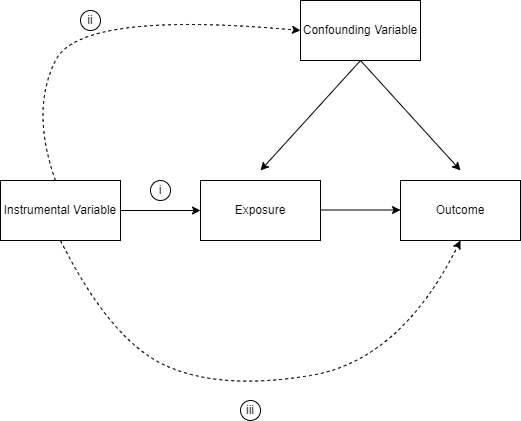


Supplementary Figure 4: Directed Acyclic Graph (DAG) of MR and its assumption. (i) genetic variants associate with the exposure of interest; (ii) there is no confounding of the SNP-outcome association; and (iii) genetic variants exert effects on the outcome only via the exposure.

*NB. The dotted lines depict no association*


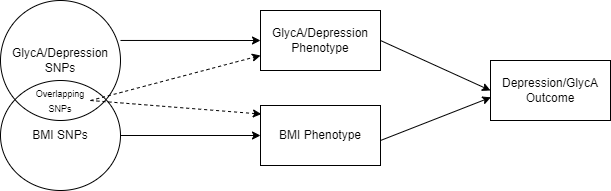


Supplementary Figure 5: Directed Acyclic Graph demonstrating the direct (solid arrows) and indirect (dashed arrows) effects in the Multivariable Mendelian Randomisation analysis


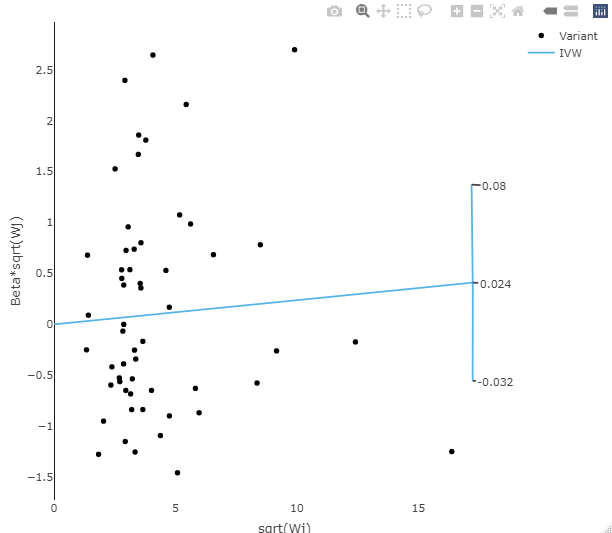


Supplementary Figure 6: Radial IVW plots of SNP-GlycA versus SNP-depressive symptom level associations, with the IVW slope.


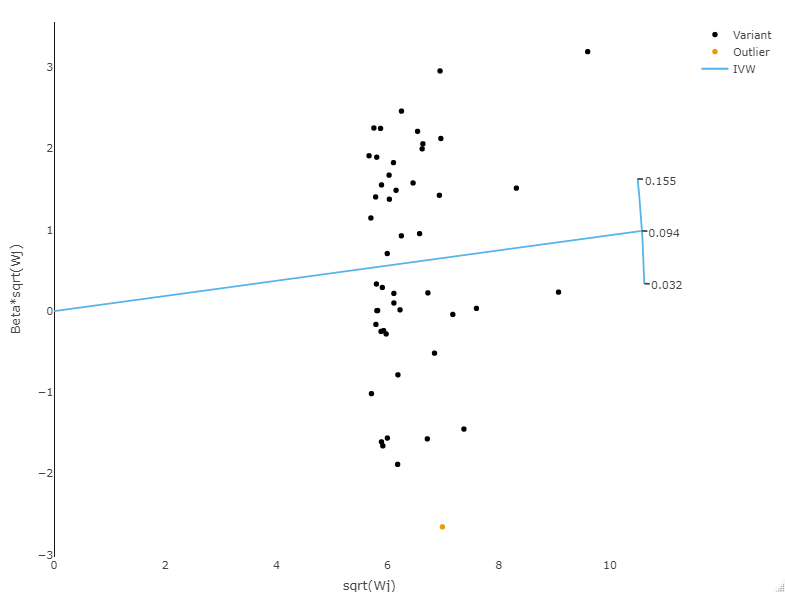


Supplementary Figure 7: Radial IVW plots of SNP-MDD versus SNP-GlycA level associations, with the IVW slope.
